# A compact stem-loop DNA aptamer targets a uracil-binding pocket in the SARS-CoV-2 nucleocapsid RNA-binding domain

**DOI:** 10.1093/nar/gkae874

**Published:** 2024-10-09

**Authors:** Morgan A Esler, Christopher A Belica, Joseph A Rollie, William L Brown, Seyed Arad Moghadasi, Ke Shi, Daniel A Harki, Reuben S Harris, Hideki Aihara

**Affiliations:** Department of Biochemistry, Molecular Biology and Biophysics, University of Minnesota, Minneapolis, MN 55455, USA; Institute for Molecular Virology, University of Minnesota, Minneapolis, MN 55455, USA; Masonic Cancer Center, University of Minnesota, Minneapolis, MN 55455, USA; Department of Biochemistry, Molecular Biology and Biophysics, University of Minnesota, Minneapolis, MN 55455, USA; Institute for Molecular Virology, University of Minnesota, Minneapolis, MN 55455, USA; Masonic Cancer Center, University of Minnesota, Minneapolis, MN 55455, USA; Department of Biochemistry, Molecular Biology and Biophysics, University of Minnesota, Minneapolis, MN 55455, USA; Institute for Molecular Virology, University of Minnesota, Minneapolis, MN 55455, USA; Masonic Cancer Center, University of Minnesota, Minneapolis, MN 55455, USA; Department of Biochemistry, Molecular Biology and Biophysics, University of Minnesota, Minneapolis, MN 55455, USA; Institute for Molecular Virology, University of Minnesota, Minneapolis, MN 55455, USA; Masonic Cancer Center, University of Minnesota, Minneapolis, MN 55455, USA; Department of Biochemistry, Molecular Biology and Biophysics, University of Minnesota, Minneapolis, MN 55455, USA; Institute for Molecular Virology, University of Minnesota, Minneapolis, MN 55455, USA; Masonic Cancer Center, University of Minnesota, Minneapolis, MN 55455, USA; Department of Biochemistry, Molecular Biology and Biophysics, University of Minnesota, Minneapolis, MN 55455, USA; Institute for Molecular Virology, University of Minnesota, Minneapolis, MN 55455, USA; Masonic Cancer Center, University of Minnesota, Minneapolis, MN 55455, USA; Institute for Molecular Virology, University of Minnesota, Minneapolis, MN 55455, USA; Masonic Cancer Center, University of Minnesota, Minneapolis, MN 55455, USA; Department of Medicinal Chemistry, University of Minnesota, Minneapolis, MN 55455, USA; Department of Biochemistry and Structural Biology, University of Texas Health San Antonio, San Antonio, TX 78229, USA; Howard Hughes Medical Institute, University of Texas Health San Antonio, San Antonio, TX 78229, USA; Department of Biochemistry, Molecular Biology and Biophysics, University of Minnesota, Minneapolis, MN 55455, USA; Institute for Molecular Virology, University of Minnesota, Minneapolis, MN 55455, USA; Masonic Cancer Center, University of Minnesota, Minneapolis, MN 55455, USA

## Abstract

SARS-CoV-2 nucleocapsid (N) protein is a structural component of the virus with essential roles in the replication and packaging of the viral RNA genome. The N protein is also an important target of COVID-19 antigen tests and a promising vaccine candidate along with the spike protein. Here, we report a compact stem-loop DNA aptamer that binds tightly to the N-terminal RNA-binding domain of SARS-CoV-2 N protein. Crystallographic analysis shows that a hexanucleotide DNA motif (5′-TCGGAT-3′) of the aptamer fits into a positively charged concave surface of N-NTD and engages essential RNA-binding residues including Tyr109, which mediates a sequence-specific interaction in a uracil-binding pocket. Avid binding of the DNA aptamer allows isolation and sensitive detection of full-length N protein from crude cell lysates, demonstrating its selectivity and utility in biochemical applications. We further designed a chemically modified DNA aptamer and used it as a probe to examine the interaction of N-NTD with various RNA motifs, which revealed a strong preference for uridine-rich sequences. Our studies provide a high-affinity chemical probe for the SARS-CoV-2 N protein RNA-binding domain, which may be useful for diagnostic applications and investigating novel antiviral agents.

## Graphical Abstract

**Graphical Abstract:**
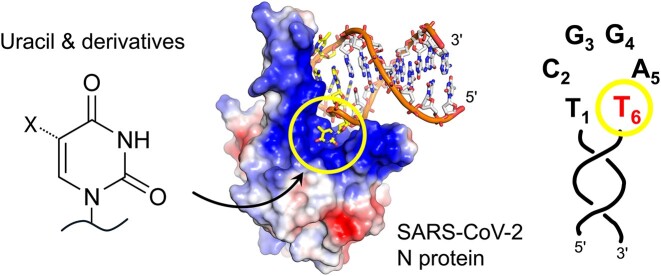

## Introduction

The large RNA genome (∼30 kb) of SARS-CoV-2 is organized in the virion as tightly packed 30–35 ribonucleoprotein (RNP) complex particles, each consisting of ∼10 copies of the viral nucleocapsid (N) protein wrapped around by RNA and arranged into a distinct cylindrical shape (1,2). N protein is one of the four SARS-CoV-2 structural proteins, along with spike (S), envelope (E) and membrane (M) proteins. Due to its abundance in virions, high expression in infected cells and relatively high sequence conservation between variants, N protein is a major target of COVID-19 rapid antigen detection tests (3–7) and is a promising vaccine candidate (8–11). Biochemical studies showed that N protein undergoes liquid–liquid phase separation (LLPS) with viral RNA (12–15). The liquid-like N-RNA condensates and an interaction between the N protein and the trans-membrane M protein are thought to play critical roles in virion assembly and budding into single membrane vesicles (16). Recent studies further showed that stem-loop-containing RNAs promote RNP formation, suggesting the importance of N protein interaction with structured RNA (17).

The 419-amino acid (aa) N protein contains an N-terminal RNA-binding domain (N-NTD) and a C-terminal dimerization domain (N-CTD) (18–20). These folded domains are flanked by intrinsically disordered regions (IDRs) of low-complexity aa sequences, which were shown to be critical for LLPS (15). The N-terminal IDR of N protein interacts with host G3BP1 to suppress stress granule assembly and promote virus production (21). The central IDR includes a ‘SR-rich’ motif, which is highly phosphorylated to modulate N-RNA condensation and potentially to regulate interaction with various host proteins including DDX1 RNA helicase (14,22,23). The central IDR also mediates interaction with the viral non-structural protein 3 (NSP3), a component of the portal complex on double-membrane vesicles (DMVs), which may facilitate the packaging of nascent viral RNA synthesized within the DMV into RNP. It has also been reported that N protein is recruited to the viral replication-transcription complex via binding to NSP3 to promote efficient viral RNA synthesis (24).

The N-NTD of SARS-CoV-2 plays a critical role in LLPS and viral RNP assembly (13). In another betacoronavirus, mouse hepatitis virus (MHV), N-NTD was also shown to bind to and melt the transcriptional regulatory sequence (TRS) RNA, a highly conserved hexanucleotide sequence motif required for subgenomic RNA synthesis (25,26). Consistent with these important functions, a point mutation in the RNA-binding motif of MHV N-NTD abolishes virus replication (26). These observations suggest that inhibition of N protein RNA-binding or targeted degradation of N protein could be a possible antiviral strategy against SARS-CoV-2 and related coronaviruses. However, there is no high-resolution structure reported for N protein in complex with any polynucleotide substrate, and selective small molecule inhibitors have yet to be developed against N protein to perturb its RNA-binding.

Here, we show that a compact stem-loop DNA aptamer binds tightly to SARS-CoV-2 N-NTD. Our X-ray crystallographic analysis demonstrates that a hexanucleotide DNA motif of the aptamer makes extensive sequence-specific contacts and engages key RNA-binding residues of N-NTD including those that form a uracil-binding pocket, making this aptamer a direct competitor of the N-RNA interaction. We also show the utility of this DNA aptamer in selective enrichment and detection of SARS-CoV-2 N protein from crude cell extracts, and in examining N-NTD interaction with RNA. Our studies provide a selective chemical probe for functional investigations of the N-RNA interaction or possible diagnostic applications and may also facilitate the development of small molecule inhibitors.

## Materials and methods

### Protein purification

A codon-optimized full-length SARS-CoV-2 N gene (encoding for N protein from the Wuhan-Hu-1 strain) was inserted between two engineered BsaI sites downstream of the T7 promoter of a modified pET-24a vector using the golden gate assembly method, along with a gene fragment for 6xHis-thioredoxin and an HRV 3C protease cleavage site between the N-terminal 6xHis-thioredoxin tag and the N protein. Similarly, codon-optimized synthetic genes for N-NTD (Pro46-Glu174) and its mutant derivatives (R92A, R107A and Y109A) and N-CTD (Ala252-Pro364) were cloned between the NdeI and BamHI sites of the pET-28a vector, with an HRV 3C protease cleavage site after the N-terminal 6xHis tag. All plasmids were verified by Sanger DNA sequencing. A single colony of *Escherichia coli* BL21(DE3) transformed with each expression plasmid was grown overnight to saturation in 25 ml ZYP-0.8G medium (27) supplemented with 100 μg ml^−1^ carbenicillin (pET-32a) or 200 μg ml^−1^ kanamycin (pET-28a). The starter culture was then used to innoculate 3 l of ZYP-5052 auto-induction medium (27) supplemented with 100 μg ml^−1^ ampicillin (pET-32a) or 200 μg ml^−1^ kanamycin (pET-28a), divided across 9 baffled 2-l shake flasks. The bacterial cells were grown at 37°C for 4 h prior to lowering the temperature to 18°C and further incubating for 20 h. The cells were pelleted, resuspended in 160 ml of 20 mM Tris–HCl pH 8.0, 1.0 M NaCl, 5 mM β-mercaptoethanol, 5 mM imidazole, and lysed by sonication. The N protein (full-length or either domain alone) was captured from centrifuged and filtered lysate using a 5 ml nickel–nitrilotriacetic acid superflow column. The column was washed extensively with the lysis buffer containing 1.0 M NaCl and the bound protein was eluted by a linear concentration gradient of imidazole from 5 to 300 mM over 165 ml. The eluted protein was treated overnight at 4°C with HRV 3C protease to remove the N-terminal thioredoxin-6xHis or 6xHis tag. The cleaved protein was concentrated by ultrafiltration and further purified by size-exclusion chromatography (SEC) on a Superdex 75 pg column operating with 20 mM Tris–HCl pH 7.4, 0.5 M NaCl. For the full-length N protein with an additional cysteine on the C-terminus (C420) used for fluorescence labeling, the final SEC buffer was supplemented with 1 mM tris(2-carboxyethyl)phosphine (TCEP). The peak fractions from SEC were verified for the presence of the target protein by SDS-PAGE, pooled, concentrated, and frozen in liquid nitrogen for storage at −80°C. The protein concentrations were determined based on UV absorbance measured on a Nanodrop 8000 spectrophotometer and the theoretical extinction coefficient from the amino acid sequence of each protein.

### Fluorescence labeling of full-length N protein

A 17.4 μl aliquot of 10 mM AZDye 488 maleimide (Fluoroprobes) in anhydrous DMSO was added to 1.0 ml of 43.2 μM SARS-CoV-2 N (C420) in 20 mM Tris–HCl pH 7.4, 500 mM NaCl, 1 mM TCEP to achieve four-fold molar excess of the dye over protein. The mixture was incubated overnight with constant inversion in the dark at 4°C. The following day, 100 μl of 20 mM Tris–HCl pH 7.4, 500 mM NaCl, 50 mM β-mercaptoethanol, 1 mM TCEP, was added and the reaction mixture was incubated on ice for 10 min to quench unreacted AZDye 488 maleimide. The labeled protein was spin-concentrated down to 500 μl and run over a Superdex 200 increase 10/300GL SEC column covered with foil in order to separate the labeled protein from the free dye. The collected protein was then spin-concentrated in an Amicon Ultra-15 10-kDa MWCO centrifugal filter. The protein was subsequently syringe-filtered through a 0.22 μm polyethersulfone membrane to remove precipitated protein, aliquoted, flash-frozen under liquid nitrogen, and stored at −80°C.

### Aptamer binding analysis by SEC

**Figure 1.**
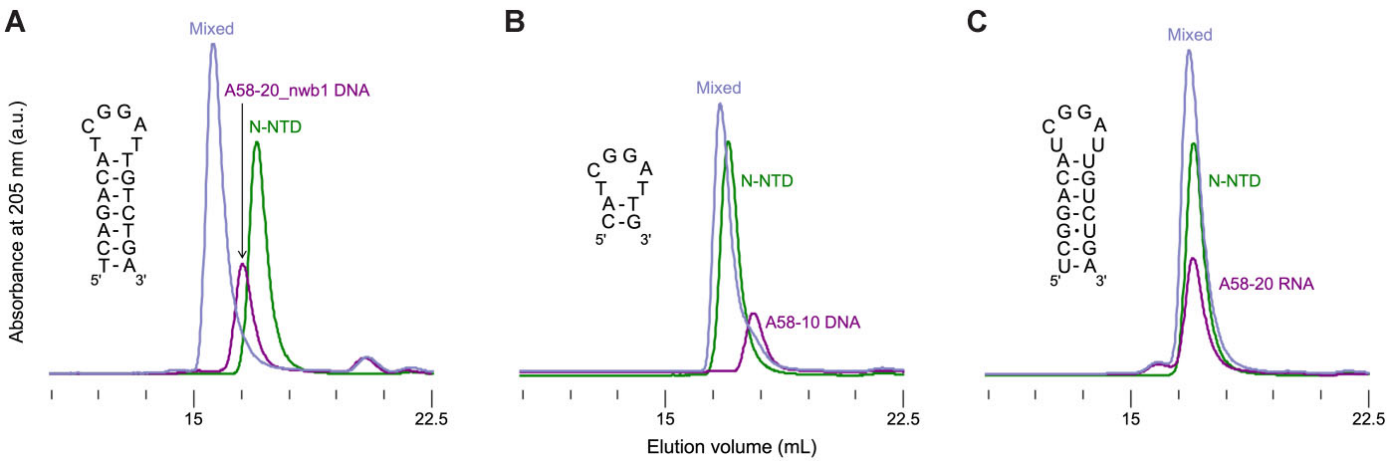
Size-exclusion chromatography (SEC) analyses of DNA or RNA aptamer binding to N-NTD. Overlaid chromatograms for N-NTD alone (green), aptamer alone (purple), and a mixed sample (slate blue) for the DNA (**A, B**) or RNA (**C**) aptamers schematically shown on the left side of each panel. UV absorbance was recorded at 205 nm. UV absorbance traces simultaneously recorded at 280 nm are shown in Supplementary Figures S3 and S4. ‘a.u.’ denotes arbitrary unit.

A 250 μl sample containing 16 μM of N-NTD or N-CTD and an approximately equimolar quantity of DNA or RNA oligonucleotide in 10 mM Tris–HCl, pH 7.4, 150 mM NaCl, and 1 mM MgCl_2_ was injected into a Superdex 200 Increase 10/300 column, operating with the same buffer and a flow rate of 0.75 ml min^−1^ at ambient temperature. Each component alone was injected under the same condition for reference. Elution of the protein and nucleic acid was detected by simultaneously monitoring UV absorption at 205, 260 and 280 nm. Overlays of the chromatograms obtained with detection at 205 nm are shown in Figure 1. Supplementary Figures S1–S4 show a complete set of chromatograms including UV traces detected at 205 and 280 nm.

### Fluorescence anisotropy

Fluorescein-labeled DNA oligonucleotides at the final concentration of 18 nM were mixed with a 2-fold serial dilution series of N-NTD or full-length N protein (final concentration: 40 or 10 μM to 9.8 nM, or 50 μM to 6.1 nM) in 10 mM Tris–HCl, pH 7.4, 150 mM NaCl, and 1 mM MgCl_2_, 0.1 mg ml^−1^ Bovine serum albumin (BSA) in a 96-well plate. The sample volume in each well was 100 μl. Fluorescence anisotropy was measured at 25°C on a Tecan Spark 10M plate reader with the excitation and emission wavelengths of 485 and 535 nm, respectively. Changes in fluorescence polarization (mP) from the control sample containing no protein were plotted against the total N-NTD or N protein concentration in the reaction. The data were fit to a custom ‘one site-specific binding with ligand depletion’ model in GraphPad Prism (*Y* = *B*_max_*(*X* − F*Y/*B*_max_)/(*K*_D_ + *X* − F*Y/*B*_max_)), *X*: total protein concentration, *F*: total fluorescence probe concentration, *Y*: fluorescence polarization after background subtraction, *B*_max_: maximum binding in the same units as *Y*, *K*_D_: dissociation constant) to determine the *K*_D_ values and 95% confidence intervals. Full-length N protein binding data were fit to the ‘Specific binding with Hill slope’ model in GraphPad Prism. For analyzing DNA/RNA binding in the competition mode, an internally fluorescein-labeled A58-20 DNA probe with a 2′-α-fluoro modification (Integrated DNA Technologies (IDT): TCGGACATC/i2FG/GA/i6-FAMK/TGTCTGA) at a concentration of 40 nM was first mixed with 400 nM of N-NTD in the same buffer as above. After a 5 min incubation at room temperature, the reactions were mixed in a 1:1 volume ratio with a 2-fold serial dilution series of unlabeled oligonucleotide (with the final concentration of 100 μM down to 780 nM for weak binding oligonucleotides, or down to 7.8 nM for stronger binding oligonucleotides) in the same buffer. The final sample volume in each well was 100 μl, containing 20 nM probe and 200 nM N-NTD. Fluorescence anisotropy was measured as above. Changes in fluorescence polarization (mP) from the control sample containing no protein but only the labeled DNA probe were plotted against the base 10 logarithm of the unlabeled oligonucleotide concentration in the reaction. The data were fitted to the ‘competitive binding—fit Ki’ model in GraphPad Prism to determine the *K*_i_ values, keeping the *K*_D_ value for the probe (13.9 nM) as a constant.

### Differential scanning fluorimetry

N-NTD at 1.0 mg ml^−1^ with or without 200 μM of various DNA or RNA oligonucleotides and 40× (final concentration) of SYPRO Orange in 10 mM Tris–HCl, pH 7.4, 150 mM NaCl, and 1 mM MgCl_2_ was heated from 20 to 95°C at a constant rate of 1°C/min in a 96-well plate on Bio-Rad CFX96 Thermal cycler. The sample volume for each well was 40 μl. Fluorescence intensity was measured with the excitation and detection wavelengths of 450–490 and 560–580 nm, respectively. The melting temperature (*T*_m_) was derived from the peak of the first derivative of the melt curve (inflection point of the melt curve).

### Biolayer interferometry

5′-Biotinylated A58-20, A58-10 and A58-58 were diluted to a concentration of 78 nM in 10 mM Tris–HCl, pH 7.4, 150 mM NaCl, 1 mM MgCl_2_, 0.05% Tween-20. Full-length SARS-CoV-2 N protein was serially diluted in the range of 25 to 1.56 nM in an identical buffer. An additional reference sample containing only buffer was added to each dilution series to remove the background during data analysis. All experiments were performed on an Octet^®^ RED384 using SAX Biosensors. Biosensor tips were pre-hydrated in pure buffer before collecting a background reading in 40 μl buffer for 60 s. The biosensor tips were then dipped into 40 μl of 78 nM biotinylated oligonucleotides for 150 s to load the aptamer onto the respective sensor for each protein dilution. After loading, the biosensor tips were dipped into 40 μl of buffer to remove unbound aptamer and to measure the background signal for 80 s. The association rate was measured by dipping the aptamer-loaded biosensor tips into 40 μl of their respective dilution of full-length SARS-CoV-2 N protein for 300 s, immediately followed by measuring the dissociation rate by transferring the biosensor tips into 40 μl of buffer. All data analysis was performed using the Octet^®^ BLI Data Analysis HT 11.1 software.

### X-ray crystallography

N-NTD was mixed with ∼1.3 times molar excess of the 20-nt DNA aptamer (A58-20) at a protein concentration of 7.6 mg ml^−1^ and dialyzed overnight at 4°C against 10 mM Tris–HCl, pH 7.4, 150 mM NaCl, 1 mM MgCl_2_. The dialysate was concentrated 2-fold by ultrafiltration and subjected to crystallization screening in sitting drop vapor diffusion mode, mixing 0.1 μl each of the complex and reservoir solutions to form the drops. Crystals of N-NTD bound to the DNA aptamer was obtained in 1 day under the condition of 0.2 M ammonium formate, 10% (w/v) polyvinylpyrrolidone, 20% (w/v) polyethylene glycol 4000. The crystals were cryo-protected by brief soaking in the reservoir solution supplemented with 20% ethylene glycol and flash-cooled by plunging in liquid nitrogen. X-ray diffraction data were collected at the NE-CAT beamline 24-ID-C of the Advanced Photon Source (Lemont, IL) and processed using XDS (28). The structure was determined by molecular replacement phasing with PHASER (29) using the previously reported SARS-CoV-2 N-NTD structure (PDB ID: 7CDZ) (19) as the search model. Iterative model building and refinement were conducted using COOT (30) and PHENIX (31). A summary of crystallographic data and model refinement statistics is shown in Table 1. Figures were generated using PyMOL (https://pymol.org/2/). The coordinates and structure factors have been deposited in the protein data bank (PDB) under the accession code 8TFD.

**Table 1.** X-ray data collection and model refinement statistics

	N-NTD/A58-20 complex
Data collection	
Space group	*P*2_1_2_1_2_1_
Unit cell dimensions	
*a, b, c* (Å)	37.80, 54.13, 98.81
Resolution (Å)	47.5−1.55 (1.61−1.55)
Total reflections	175 455 (5151)
Unique reflections	27 781 (1655)
Completeness (%)	91.87 (55.38)
Multiplicity	6.3 (3.1)
*R*_merge_	0.0735 (0.425)
*R*_meas_	0.0798 (0.505)
*R*_pim_	0.0306 (0.265)
*I / σI*	16.93 (1.88)
CC_1/2_	0.934 (0.804)
Refinement	
Resolution (Å)	47.48–1.55 (1.61–1.55)
No. reflections	27 753 (1653)
*R*_work_/*R*_free_	0.159/0.191
No. atoms	1654
Protein	1397
Ligand/ion	20
Water	237
*B*-factor (Å^2^)	28.53
Protein	24.83
Ligand/ion	37.90
Water	35.78
R.m.s. deviations	
Bond lengths (Å)	0.013
Bond angles (°)	1.26

Statistics for the highest-resolution shell are shown in parentheses.

### DNA aptamer-mediated N-protein pull-down from spiked *E. coli* lysate

Untransformed BL21(DE3) *E. coli* was grown overnight in 500 ml LB-medium at 37°C. The culture was split into two halves and centrifuged at 4000 × g for 30 min at 4°C. The supernatant was removed, and the pellets were stored at −20°C. For each experiment, a single pellet tube was thawed and resuspended in *E. coli* lysis buffer (10 mM Tris–HCl, 150 mM NaCl, 0.05% Tween20, 1.0 mM EDTA, 1× BugBuster (MilliporeSigma)). 200 μl RNase A (20 mg ml^−1^, Invitrogen) was added, and the mixture was incubated at 37°C for 30 min to allow for complete lysis of cells. Following this, the lysate was centrifuged at 18 000 × g for 10 min at 4°C, and 4 ml of the supernatant was carefully removed to avoid disturbing the insoluble pellet. The supernatant was transferred to a clean tube and supplemented with 19.2 μl of 6.3 mg ml^−1^ AZDye 488-labeled full-length N protein. The spiked lysate mixture was thoroughly mixed by inverting the tube before being incubated on ice for 10 min. Following this, the lysate was again centrifuged at 18 000 × g for 10 min at 4°C to pellet any insoluble proteins.

While preparing the spiked lysate, 160 μl (1.6 mg) M-280 Streptavadin coated magnetic Dynabeads (Invitrogen) were transferred to a 1.5 ml centrifuge tube and washed twice with 1 ml B/W buffer (5 mM Tris–HCl pH 7.4, 1.0 M NaCl, 0.5 mM EDTA, 0.1% Tween-20) for 5 min at room temperature. The B/W buffer was entirely removed using suction before resuspending the beads in 160 μl of fresh B/W buffer. The washed beads were mixed by gently flicking until homogeneous and split into two 1.5 ml tubes containing 80 μl (0.6 mg) resuspended beads. An additional 400 μl of B/W buffer was then added to each tube, followed by 20 μl ultra-pure water for the control tube, or 20 μl 1.0 mM (130.82 ng) 5′-biotinylated A58-20 DNA aptamer for the aptamer tube. The control and aptamer tubes were then placed on a rotating shaker for 1 hour at room temperature to facilitate binding. The beads were then washed 3 times with 1 ml ice-cold protein binding buffer (20 mM Tris–HCl pH 7.4, 150 mM NaCl 1.0 mM EDTA, 0.1% Tween-20). After washing, the buffer was completely removed via suction and 610 μl spiked *E. coli* lysate was added to both the control and aptamer beads. Before incubation, 10 μl of lysate was removed from both control and aptamer tubes and saved as the ‘pre-binding’ controls. The tubes were gently mixed by flicking to fully resuspend the beads. Both tubes were then placed on a rotating shaker for 2 h at 4°C. After binding, the lysate was stored as the ‘post-binding’ sample. The beads were then resuspended in 1 ml of cold protein binding buffer and placed on a rotating shaker for 10 min at 4°C. The beads were then magnetized, and the supernatant was stored as the ‘wash-1' sample. This process was then repeated 2× additional times. After washing, all residual buffer was removed via suction and the beads were resuspended in 30 μl 10× SDS running buffer (250 mM Tris base, 1.9 M glycine, 1% SDS, pH 8.3). The beads were fully resuspended by pipette before incubating at 95°C for 10 min. Beads were then magnetized, and the eluate was stored. For SDS-PAGE, all samples were diluted 2:3 in 2× SDS-PAGE loading buffer (100 mM Tris–HCl pH 6.8, 4% SDS, 0.8% bromophenol blue, 20% glycerol) and incubated at 95°C for 10 min. Samples were loaded onto a Mini-PROTEAN TGX gel (Bio-Rad) and run at 200 V for 35 min. Gels were then scanned using an Amersham Typhoon 9500 imager and Amersham Typhoon Scanner Control Software 2.0.0.6 with the built-in Cy2 scanning method. After fluorescence scanning, gels were stained with Coomassie Brilliant Blue G-250 (G-Biosciences) for 2 h at room temperature. Gels were imaged with the Gel Doc EZ platform and Image Lab software (Bio-Rad).

### DNA aptamer-mediated N protein isolation/enrichment from 293T cells

160 μl (1.6 mg) M-280 Streptavadin coated magnetic Dynabeads (Invitrogen) were transferred to a 1.5 ml centrifuge tube and washed twice with 1 ml B/W buffer (5 mM Tris–HCl pH 7.4, 1.0 M NaCl, 0.5 mM EDTA, 0.1% Tween-20) for 5 min at room temperature. The B/W buffer was entirely removed using suction before resuspending the beads in 160 μl of fresh B/W buffer. The washed beads were mixed by gently flicking until homogeneous and split into two 1.5 ml tubes containing 80 μl (0.6 mg) resuspended beads. An additional 400 μl of B/W buffer was then added to each tube, followed by 20 μl ultra-pure water for the control tube, or 20 μl 1.0 mM (130.82 ng) 5′-biotinylated A58-20 DNA aptamer for the aptamer tube. The control and aptamer tubes were then placed on a rotating shaker for 1 h at room temperature to facilitate binding. The beads were then washed 3 times with 1 ml ice-cold 293T protein binding/lysis buffer (20 mM Tris–HCl pH 7.4, 150 mM NaCl 2.0 mM EDTA, 1.0 mM TCEP, 1% NP-40, 1× Roche EDTA-free protease inhibitor tablet / 10 ml). Approximately 16 million N-GFP expressing 293T cells were thawed and resuspended in 1.5 ml binding/lysis buffer and placed on a rotating shaker at 4°C for 2 h to facilitate lysis. After lysis, 50 μl was saved as the ‘raw lysate’ control. The cells were centrifuged at 18 000 × g for 10 min at 4°C and another 50 μl was collected as the ‘pre-binding’ control. The cell lysate was then split into control and aptamer tubes, with each receiving 700 μl of lysate. The tubes were gently mixed by flicking to fully resuspend the beads. Both tubes were then placed on a rotating shaker for 2 h at 4°C. After binding, the lysate was stored as the ‘post-binding’ sample. The beads were then resuspended in 1 ml of cold protein binding/lysis buffer and placed on a rotating shaker for 10 min at 4°C. The beads were then magnetized, and the supernatant was stored as the ‘wash-1' sample. This process was then repeated 2× additional times. After washing, all residual buffer was removed via suction and the beads were resuspended in 30 μl 10× SDS running buffer. The beads were fully resuspended by pipette before incubating at 95°C for 10 min. Beads were then magnetized, and the eluate was stored. SDS-PAGE was performed as described above.

### N protein isolation/enrichment western blots

All samples were diluted 2:3 in 2× SDS-PAGE loading buffer and incubated at 95°C for 10 min. Samples were loaded onto a Mini-PROTEAN TGX gel (Bio-Rad) and run at 90 V for 15 min, followed by running at 150 V for 1 h. Membrane transfer was performed at 80 V for 1.5 h at 4°C. The membranes were then blocked in PBS with 0.1% Tween-20 and 4% fat-free milk. Primary antibody incubation was performed with rabbit anti-SARS-CoV-2 Nucleocapsid GTX635686-01 (GeneTex) at a dilution of 1:5000 for 1.5 h at room temperature. The membranes were washed for 5 min in PBS with 0.1% Tween-20 at room temperature 6 times, followed by incubation with a secondary antibody, goat anti-rabbit 680RD 925–68071 (LI-COR) at a dilution of 1:20 000 at room temperature for 1.5 h. The membranes were washed again for 5 min in PBS with 0.1% Tween-20 6 times. Membranes were scanned using an Amersham Typhoon imager and Amersham Typhoon Scanner Control Software 2.0.0.6 with the ‘IR Short’ built-in method.

### Sandwich ELISA

A 3′-biotinylated DNA stem-loop A58-20 aptamer with a poly-dA35 linker on the 3′ side, or its loop variant T6-to-A (60 pmol), diluted in Wash Buffer (25 mM Tris–HCl, 150 mM NaCl, 0.1% BSA, 0.05% Tween-20; pH 7.2), was immobilized on a Streptavidin Coated High Binding Capacity plate (Pierce 15501) at room temperature with gentle rocking for 2 h. The plate was washed 3 times, and then 100 μl of the lysate of 293T cells expressing N-GFP or its point mutant derivative, N-GFP (Y109A), was added to each well with serial dilutions in Wash Buffer. After 1 hour of incubation, the plate was washed 3 times with the Wash Buffer. The captured proteins were detected with an N protein antibody AS41 (ACROBiosystems NUN-S41, 1:5000 dilution in Wash Buffer) and a horseradish peroxidase (HRP)-conjugated secondary antibody (Jackson ImmunoResearch 109035088, 1:10000 dilution in Wash Buffer). The signal was visualized with 1-Step Ultra TMB-ELISA Substrate Solution (Thermo Scientific 34028) and quantified using a Tecan Spark microplate reader at 450 nm. The N protein concentration in the lysate sample was estimated using a standard curve generated for a dilution series of N protein with known concentration, separately expressed and purified from *E. coli*.

### Plasmid generation and transfection

All plasmids for expression in mammalian cells were generated by traditional molecular cloning using restriction enzyme digestion and ligation by T4 DNA ligase (New England BioLabs, M0202L). The cDNA for SARS-CoV-2 N was synthesized as codon-optimized gBlocks from IDT based on the available amino acid sequence (Addgene 141391). The sequence was amplified by PCR, digested using EcoRI and AgeI, and ligated into a pcDNA3.1 upstream of an in-frame GFP coding sequence and the resulting colonies were sequence verified by Sanger sequencing. The Y109A mutation was introduced by site-directed mutagenesis and sequence verified by Sanger sequencing. To express SARS-CoV-2 N and its derivatives in mammalian cells, 293T cells were plated in a 10 cm dish at a density of 3 × 10^6^ and allowed to adhere overnight. The following day, 2 μg of plasmids were transfected into cells using TransIT-LT1 transfection reagent (Mirus MIR 2300) as per the manufacturer's protocol. At 48 h post-transfection, cells were trypsinized, resuspended in PBS, pelleted at 500 × g for 5 min, and stored frozen until use.

## Results

### DNA aptamer targets N-NTD

Various RNA and DNA sequences, including *in vitro* selected aptamers and stem-loop motifs derived from viral genomes, have been reported to bind to coronavirus N proteins (26,32–36). We tested whether these sequences bind to an isolated structural domain of SARS-CoV-2 N protein, by mixing chemically synthesized oligonucleotides with purified N-NTD or N-CTD and monitoring their co-migration in SEC (Supplementary Figures S1, S2). We found that two 58-nucleotide (nt) DNA aptamers, A58 and A61, which were identified by Zhang et al. using the systematic evolution of ligands by exponential enrichment (SELEX) approach against full-length SARS-CoV-2 N protein (36), form stable complexes selectively with N-NTD. Both these DNA aptamers contain stem-loop elements centered on a common hexanucleotide loop (5′-TCGGAT-3′), suggesting that this motif may be involved in N-NTD binding. To investigate this possibility, the binding of truncated A58 aptamers was tested. A 20-nt stem-loop A58-20 (5′-TC**G**GACATCGGATTGTC**T**GA-3′), or its derivative with a G•T wobble pair (the 3rd and 18th nucleotides shown in bold) changed to an A–T pair (A58-20_nwb1: 5′-TC**A**GACATCGGATTGTC**T**GA-3′), formed a stable complex with N-NTD, which eluted earlier than either the protein or DNA alone in SEC (Figure 1A, Supplementary Figure S3). By incrementally truncating the base-paired stem, we found that the central 10-nt (A58-10: 5′-CATCGGATTG-3′) is sufficient to form a stable complex with N-NTD separable in SEC at ambient temperature (Figure 1B, Supplementary Figure S3). However, further trimmed aptamers (*e.g*. A58-8: 5′-ATCGGATT-3′) or those with nucleotide substitutions in the hexanucleotide loop did not show complex formation (Supplementary Figure S4). In addition, an RNA counterpart of A58-20 (A58-20_RNA: 5′-UCGGACAUCGGAUUGUCUGA-3′) failed to form a stable complex with N-NTD (Figure 1C, Supplementary Figure S4). These results show that the hexanucleotide DNA motif in a stem-loop context plays a key role in N-NTD binding.

### Affinity of DNA aptamer to N-NTD

**Figure 2.**
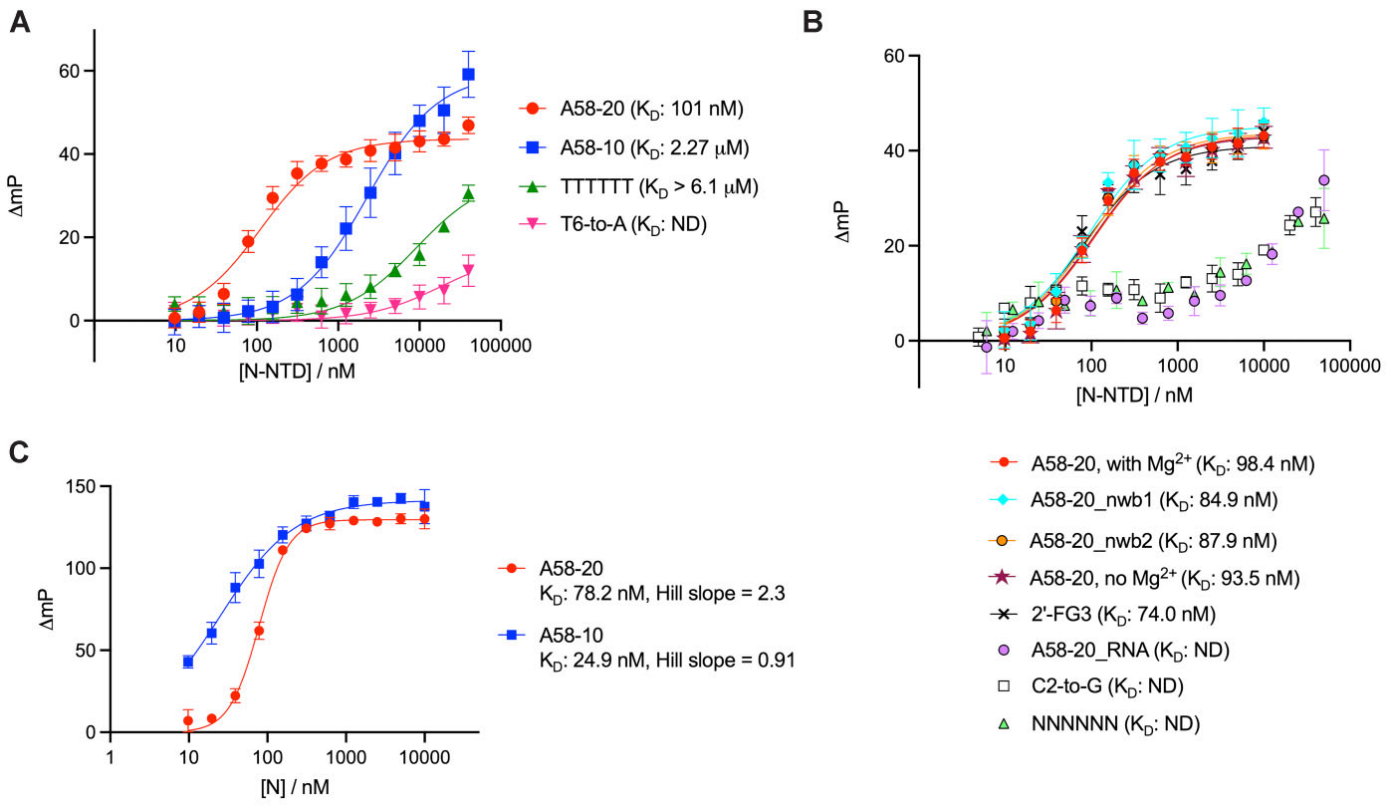
Fluorescence anisotropy analyses of aptamer binding to N-NTD or full-length N protein. (**A, B**) Binding of A58-20 DNA or its variants, including those designed based on the structural information presented below (Figure 4), to N-NTD. **(C)** Binding of A58-20 and A58-10 DNA to full-length N protein. Changes in fluorescence polarization (mP) from the control sample containing no protein were plotted against the total protein concentration in the reaction. ND, not determined. Schematics of the DNA aptamers are shown in Figure 3A. Data points are the mean ± standard deviation of at least six (A) or three (B, C) technical replicates. All oligonucleotides used in the experiments shown in this figure were fluorescently labeled on the 3′ end. The *K*_D_ values determined using the FA assay are summarized in Supplementary Table S1.

**Figure 3.**
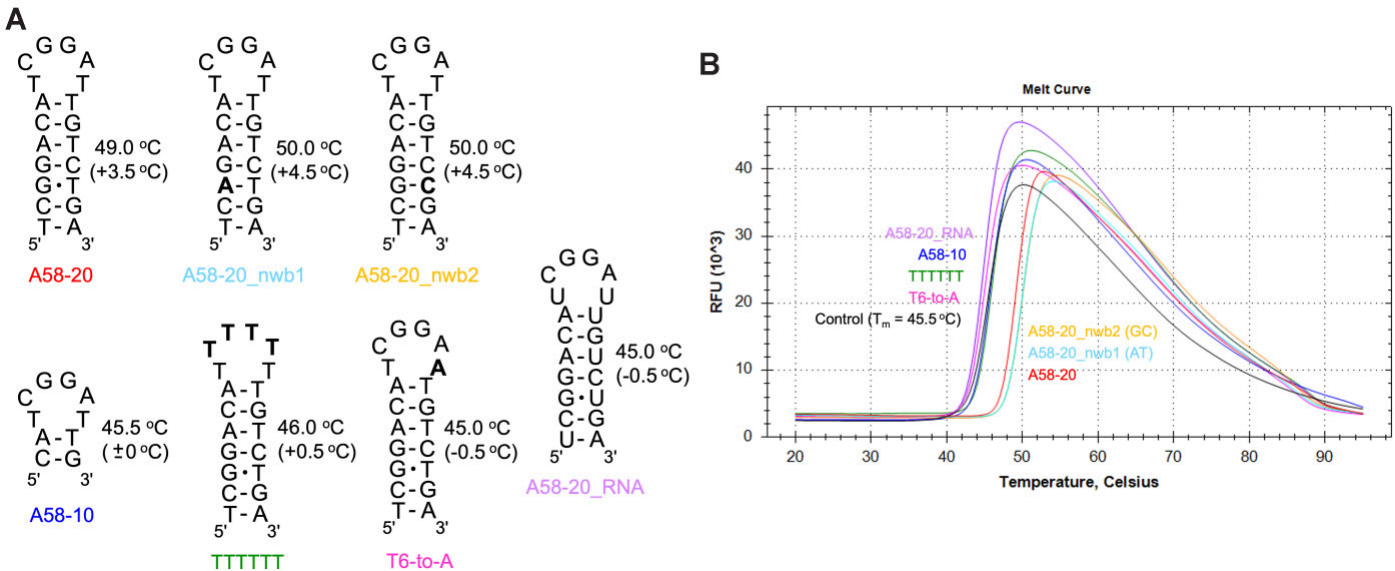
DSF analyses of the stabilizing effect of DNA/RNA aptamers on N-NTD. (**A**) Schematics of various stem-loop aptamers. Sequence modifications from the parental A58-20 are highlighted in bold. For each aptamer, the T_m_ value of N-NTD in its presence is shown on the right (the shift of *T*_m_ from that for N-NTD alone is shown in parenthesis). (**B**) Melt curves of N-NTD in the presence of indicated aptamers. The control sample (black trace) contained no aptamer. Identical *T*_m_ values were obtained in duplicate experiments, from which one melt curve for each sample is shown.

The affinity of the DNA stem-loop toward isolated N-NTD was measured using fluorescence anisotropy (FA). A58-20 DNA with a 3′ fluorescein (FAM) modification bound N-NTD with an equilibrium dissociation constant (*K*_D_) of 101 nM (95% CI: 91.4–110 nM) (Figure 2A, Supplementary Table S1). Consistent with the SEC results, changing the G•T wobble pair in the stem to either an A–T or G–C pair did not significantly alter the affinity; A58-20_nwb1 and A58-20_nwb2 bound N-NTD with *K*_D_ values of 84.9 nM (95% CI: 65.5–109 nM) and 87.9 nM (95% CI: 72.0–107 nM), respectively (Figure 2B). A58-10 with a very short 2 bp stem also showed binding, albeit with lower affinity (*K*_D_: 2.27 μM, 95% CI: 1.91–2.69 μM), which likely reflects a greater entropic cost of binding as a stem-loop (Figure 2A). Changing the central 4 bases of A58-20 to all thymines, which makes the hexanucleotide loop sequence TTTTTT, largely abolished the binding (*K*_D_ > 6.1 μM) (Figure 2A and see Figure 3A for schematics of the variants). Interestingly, a single nucleotide substitution at the 6th position of the hexanucleotide loop (T6-to-A) had an even more severe effect on the interaction with N-NTD (*K*_D_ not determined due to low affinity), underscoring the importance of the DNA sequence of the hexanucleotide loop and, in particular, the last T6 base (Figure 2A). Other variants of A58-20, with a single nucleotide substitution at the second position of the hexanucleotide loop (C2-to-G) or a fully randomized loop (NNNNNN) also showed weaker binding, with a somewhat anomalous pattern of fluorescence polarization increase as a function of protein concentration (Figure 2B). Similar behavior was observed for A58-20_RNA (Figure 2B), which showed weak binding consistently with the lack of co-elution with N-NTD in SEC (Figure 1C, Supplementary Figure S4). In addition, we analyzed the binding of full-length N protein to A58-20 and A58-10 in the FA assay (Figure 2C). Full-length N protein bound with a similar apparent affinity as N-NTD to A58-20, although the data exhibited positive cooperativity (*K*_D_: 78.2 nM, 95% CI: 73.9–82.8 nM; Hill slope = 2.3, 95% CI: 2.0–2.6). On the other hand, full-length N protein bound with a higher affinity than N-NTD to A58-10 (*K*_D_: 24.9 nM, 95% CI: 21.8–28.5 nM) without a strong sign of cooperativity (Hill slope = 0.91, 95% CI: 0.79–1.0). Thus, the residues outside NTD affect the binding of full-length N protein to these DNA aptamers in a DNA length-dependent fashion.

### DNA aptamer stabilizes N-NTD

Results of the binding analyses by SEC and FA are corroborated by a thermal stability measurement using differential scanning fluorimetry (DSF), which showed that binding of the 20-nt DNA aptamers stabilizes N-NTD (Figure 3). The presence of a saturating concentration (200 μM) of A58-20 shifted the melting temperature (*T*_m_) of N-NTD from 45.5 to 49.0°C. The *T*_m_ shift was even greater (Δ*T*_m_ = +4.5°C) when the G•T wobble pair in the stem was changed to either an A–T or G–C pair. The poorly binding loop variants, TTTTTT and T6-to-A, elicited marginal *T*_m_ shift with a Δ*T*_m_ of +0.5 and –0.5°C, respectively. Similarly, A58-20_RNA gave a Δ*T*_m_ of –0.5°C, consistent with its low affinity. Lastly, the shorter stem variant A58-10 showed little stabilizing effect (Δ*T*_m_ = 0°C), likely reflecting the low thermal stability of this stem-loop itself. The FA and DSF data together suggest that the base pairing of the stem, in addition to the sequence of the hexanucleotide loop, is important for the binding of A58-20 DNA to N-NTD.

### N-NTD engages the hexanucleotide loop

**Figure 4.**
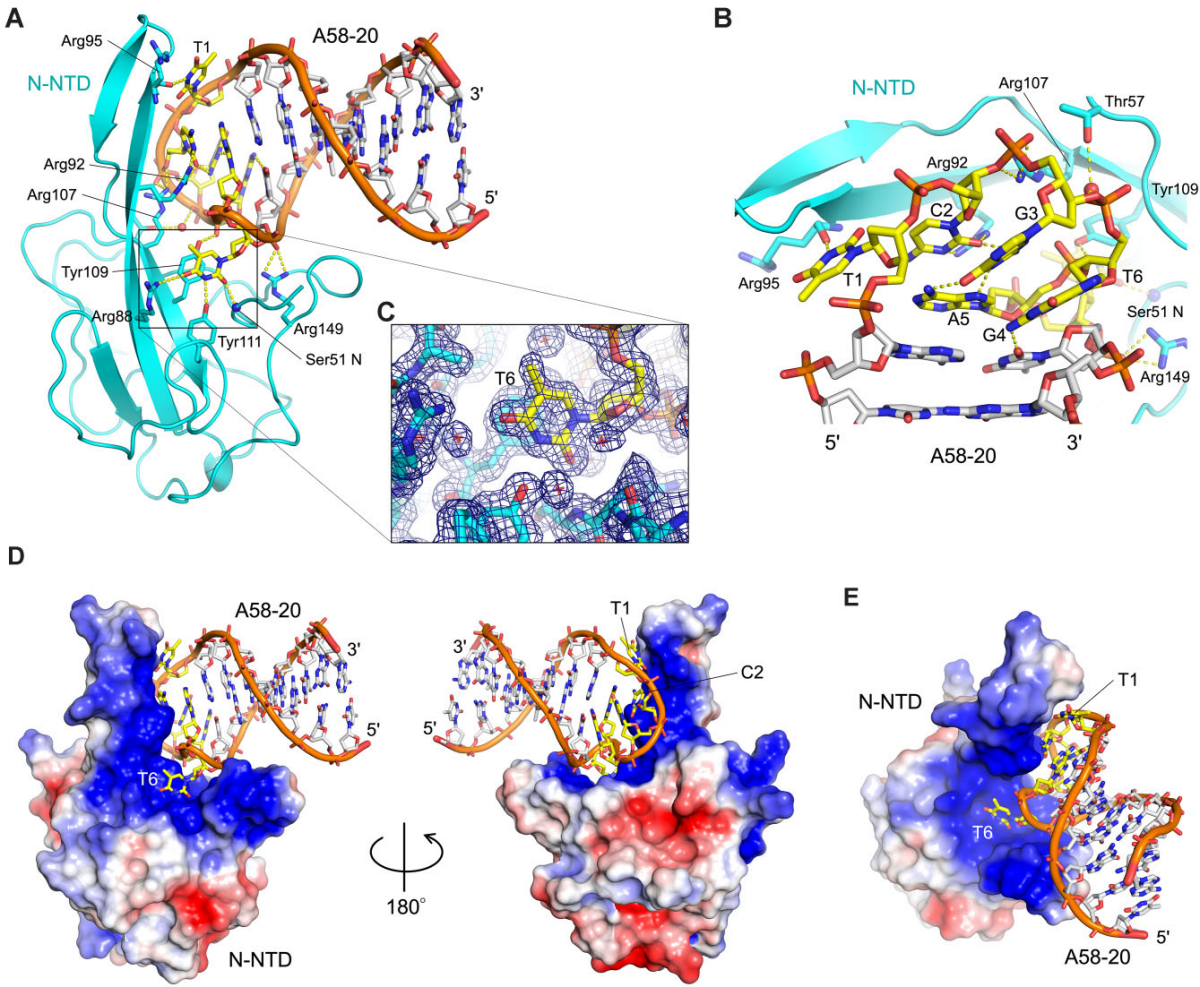
Crystal structure at 1.55-Å resolution of the A58-20 DNA aptamer in complex with N-NTD. (**A**) Cartoon representation of the complex. The hexanucleotide loop is highlighted in yellow. (**B**) A zoom-in view of the hexanucleotide loop from the backside in (A). (**C**) The flipped-out 6th thymine base (T6) of the hexanucleotide loop, fit in a uracil-binding pocket and stacked on Tyr109. The 2mFo-DFc electron density map contoured at 1.0 σ above the mean level is shown as a blue mesh. The map for the whole complex is shown in Supplementary Figure S5. Small red spheres and crosshairs represent water molecules. Yellow dashed lines indicate hydrogen bonds. (**D**) Molecular surface of N-NTD bound to A58-20 DNA viewed from two opposite orientations, colored according to the electrostatic potential (–2.5 kT/e in red to +2.5 kT/e in blue). (**E**) Same as (D), in a different orientation.

**Figure 5.**
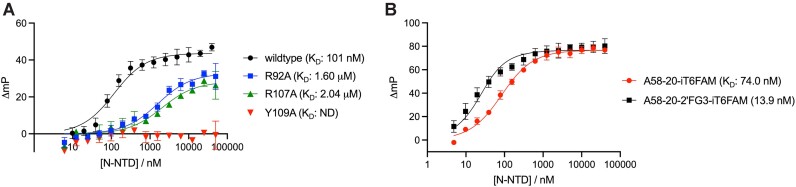
Structure-guided modifications modulate the aptamer affinity. (**A**) The binding of wildtype and three mutant derivatives of N-NTD to the 3′ FAM-labeled A58-20 DNA, as monitored by FA. The Y109A substitution completely abolishes binding. (**B**) The binding of wildtype N-NTD to an A58-20 DNA aptamer internally FAM-labeled on the T6 base, with or without a 2′-α-fluoro modification on G3. Data points are the mean ± standard deviation of three replicates. ND, not determined.

To understand the mechanism of sequence-specific DNA binding, we determined a crystal structure of N-NTD in complex with A58-20. The structure was refined to 1.55-Å resolution with excellent model quality and fit to experimental data (*R*_work_/*R*_free_ = 15.9/19.1%, Table 1, Supplementary Figure S5). The A58-20 aptamer binds to a highly positively charged concave surface of N-NTD, which was shown by earlier NMR studies to be involved in RNA-binding (Figure 4A, D, E) (37). Consistent with the biochemical observations above, N-NTD engages the hexanucleotide loop of the aptamer, whereas the flanking sequences form a double-stranded stem pointed away from the protein (Figure 4A). The 5′-TCGGAT-3′ sequence folds into a compact loop stabilized by a network of intra-molecular hydrogen bonds, including a non-canonical G-A base-pair (Figure 4B). N-NTD makes extensive backbone as well as base contacts. The thymine base at the first position (T1) is hydrogen-bonded to the Arg95 backbone oxygen atom. The cytosine base at the 2nd position (C2) forms bidentate hydrogen bonds with Arg92, consistent with the reduced affinity of the C2-to-G variant (Figure 2B). The thymine base at the 6th position (T6) is flipped out of the loop and π-stacked against Tyr109, where it is stabilized by hydrogen bonding to the main chain nitrogen atom of Ser51 and the side chains of Arg88 and Tyr111 (Figure 4C), explaining the critical role of this base and the loss of affinity for the T6-to-A variant (Figure 2A). Tyr109 is also hydrogen-bonded to the backbone phosphate and thus appears to be a key residue in the interface. Notably, a Y109A amino acid substitution was reported to abolish RNA-mediated LLPS of SARS-CoV-2 N protein (13). Thus, the DNA aptamer engages an essential RNA-binding residue of N-NTD. The binding of N-NTD to the compact stem-loop is also supported by phosphate backbone contacts made by Arg107 and Arg149 (Figure 4A). N-NTD mutant derivatives R92A and R107A showed ∼20-fold weaker affinity to A58-20 DNA than wildtype N-NTD, whereas Y109A amino acid substitution completely abolished the binding (Figure 5A). The results of this structure-guided mutagenesis experiment corroborate the crystallographic observations and highlight, in particular, the importance of the interaction made with the T6 base.

Even though 1 mM MgCl_2_ was included in the N-NTD/DNA complex sample subjected to crystallization, the crystal structure of N-NTD bound to A58-20 did not show a bound metal ion. The binding behavior of N-NTD to A58-20 monitored by FA in the presence of 1 mM MgCl_2_ was indistinguishable from that observed in the presence of 1 mM ethylenediaminetetraacetic acid (EDTA) and no Mg^2+^, confirming that Mg^2+^ does not play a role in the binding (Figure 2B). An additional observation about the conformation of A58-20 DNA bound to N-NTD is that the 3^rd^ nucleotide (G3) of the hexanucleotide loop involved in the G-A base-pair takes the RNA-like C3′-endo sugar pucker (Figure 4B). Consistently, A58-20 with a substitution of 2′-deoxy-2′-α-fluoroguanosine for G3, which favors the C3′-endo conformation (38,39), was fully competent in binding N-NTD (2′-FG3; Figure 2B). The crystal structure also showed that, although the T6 base makes critical protein contacts, its methyl group is pointed toward the solvent and makes no protein contacts (Figure 4C, E). We took advantage of this observation and designed an alternative probe for FA, in which fluorescein is attached to 5th position of the pyrimidine ring of T6 via click chemistry (A58-20-iT6FAM). A58-20-iT6FAM bound to N-NTD with a *K*_D_ of 74.0 nM (95% CI: 64.9–84.1 nM), comparable to the binding of A58-20-3′FAM above, while generating greater polarization signals (ΔmP) upon N-NTD binding (Figure 5B). An addition of the 2′-α-fluoro modification on G3 (A58-20-2′FG3-iT6FAM) improved the affinity for N-NTD to a *K*_D_ of 13.9 nM (95% CI: 10.0–18.8 nM), which represents the highest affinity of all oligonucleotide probes tested (Figure 5B). The *K*_D_ values determined using the FA assay are summarized in Supplementary Table S1.

### DNA aptamer binds avidly to full-length N protein

**Figure 6.**
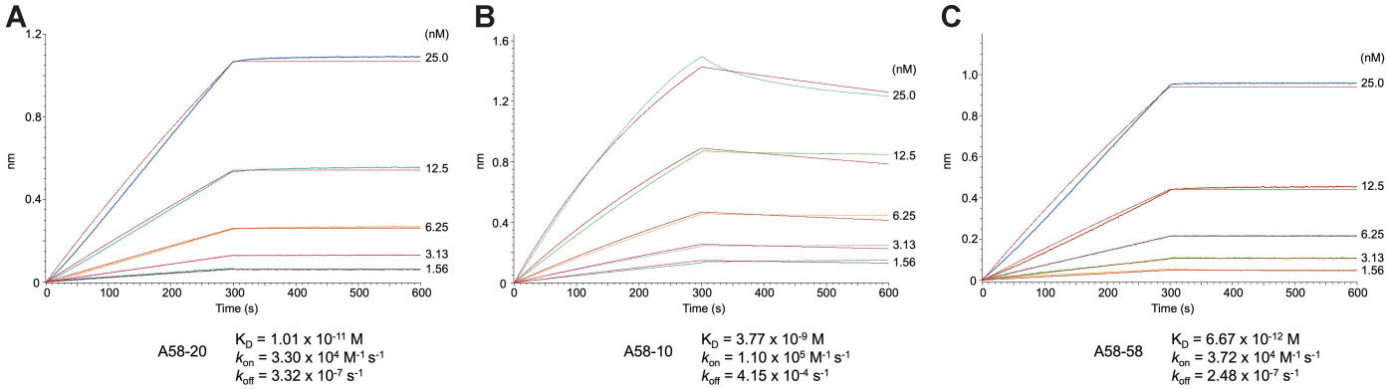
Avid binding of full-length N protein to DNA aptamers. BLI sensorgrams showing the association and dissociation kinetics of full-length N protein to the immobilized A58-20 (**A**), A58-10 (**B**), and the parental 58-mer A58 DNA aptamer (**C**). Best fit curves to the single-site binding model are shown in red. The protein concentrations tested were 1.56, 3.13, 6.25, 12.5 and 25.0 nM. Representative results of four (A) or three (B, C) replicates are shown.

Next, we used biolayer interferometry (BLI) to analyze the interaction between full-length N protein and 5′-biotinylated A58-20 or A58-10 DNA aptamer immobilized on the streptavidin-coated biosensor surface. As N protein forms a stable homodimer via N-CTD (19,20), we reasoned that full-length N protein should bind more tightly to the DNA aptamer-coated surface than does the isolated N-NTD due to higher avidity. Full-length N indeed showed very slow dissociation (3.32 × 10^−7^ s^−1^) and a correspondingly high affinity for A58-20, with the *K*_D_ of 10.1 pM (Figure 6A). We analyzed in parallel the binding of the parental 58-nt A58 aptamer and obtained a comparable *K*_D_ of 6.67 pM (Figure 6C), consistent with the notion that regions outside the stem-loop represented by A58-20 play minor if any, roles in N-NTD binding. Of note, these *K*_D_ values are much smaller (i.e. higher affinity) than those reported originally by Zhang *et al.* for the binding of full-length N protein to the 58-nt A58 and A61 DNA aptamers, which were 0.70 and 2.74 nM, respectively, as determined using surface plasmon resonance (SPR) (36). The difference could be attributable to different methods used. Full-length N protein also showed tight binding to A58-10 in BLI, with a *K*_D_ of 3.77 nM (Figure 6B).

### Aptamer-mediated pull-down of N protein

**Figure 7.**
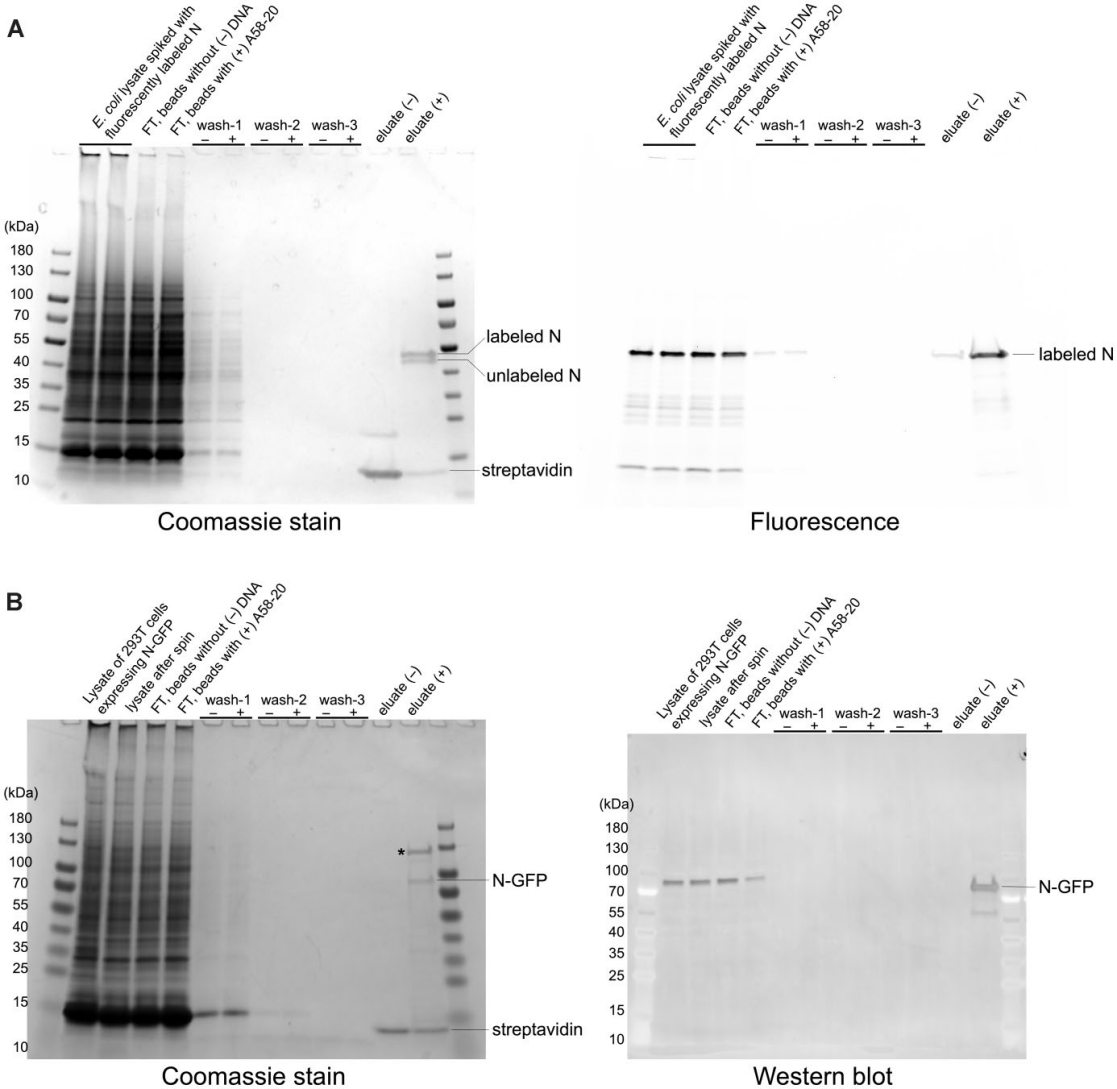
DNA aptamer-mediated selective pull-down of SARS-CoV-2 N protein. (**A**) Pull-down from an *E. coli* lysate spiked with AZDye 488-labeled N protein. The SDS-PAGE gel on the left was stained by Coomassie blue for the total protein, whereas the gel on the right was scanned for fluorescence. Every two lanes show a comparison of results with Streptavidin-coated magnetic beads without (–) and with (+) the biotinylated A58-20 DNA aptamer. ‘FT’ denotes flow through. The Coomassie-stained gel shows that both AZDye 488-labeled and residual unlabeled N protein were pulled down. Based on a fluorescence intensity measurement, 2.1 and 0.16% of the total labeled protein was recovered in (+) and (–) eluate, respectively, in this experiment. (**B**) Pull-down from the lysate of 293T cells expressing N-GFP. The SDS-PAGE gel on the left was stained by Coomassie blue for the total protein. Immunoblot with an anti-N monoclonal antibody is shown on the right. Representative results of two (A) or three (B) replicates are shown.

We then tested the selectivity of the A58-20 DNA aptamer. An *E. coli* lysate was spiked with a full-length SARS-CoV-2 N protein, which had been previously purified and fluorescently labeled using an extra Cys residue added to the C-terminus, and the crude mixture was subjected to pull-down with streptavidin magnetic beads conjugated to the biotinylated A58-20 aptamer. SDS-PAGE gels stained with Coomassie blue or scanned for fluorescence show that only the N protein from this crude protein mixture bound to the DNA aptamer-conjugated beads and survived extensive washes, whereas the streptavidin beads without the aptamer showed negligible binding (Figure 7A). We further showed that the A58-20 aptamer immobilized on streptavidin magnetic beads, but not the beads without DNA, can pull down and enrich N protein from the lysate of 293T cells expressing an N-GFP construct (Figure 7B). These results demonstrate high selectivity of the aptamer binding. Curiously, we reproducibly observed an endogenous ∼130 kDa protein from the cell lysate that also showed selective pull-down and enrichment (marked by an asterisk in Figure 7B). Further studies are warranted to investigate the identity and the mechanism of binding of this human protein.

### Aptamer-mediated N protein detection

**Figure 8.**
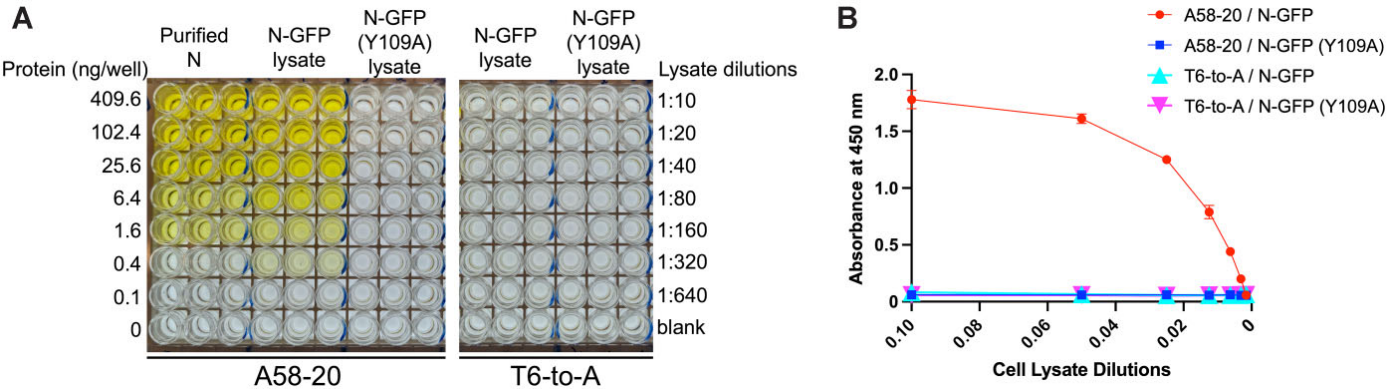
Sandwich ELISA assay detecting SARS-CoV-2 N protein from the lysate of 293T cells expressing N-GFP or N-GFP (Y109A) protein. (**A**) A photograph of ELISA plates from triplicated experiments for the A58-20 DNA aptamer (left) or its T6-to-A variant (right). The amount of the purified N protein from *E. coli* diluted in the wash buffer used as a standard is shown on the left. Lysates were diluted 10 (0.10) to 640-fold (0.00156). The graph in (**B**) shows the quantitation of the ELISA data for the lysate samples.

Given the high-affinity and selective binding of the DNA stem-loop motif to SARS-CoV-2 N protein, we further explored its utility in the detection of N protein from crude samples in a sandwich enzyme-linked immunosorbent assay (ELISA). The biotinylated A58-20 DNA aptamer was immobilized on the streptavidin-coated 96-well plate surface, and the lysate of 293T cells expressing N-GFP or its point mutant derivative, N-GFP Y109A, was added to each well with serial dilutions. After extensive washing, the captured proteins were detected with an anti-N protein human IgG1 AS41, which specifically binds to N-CTD, and a horseradish peroxidase (HRP)-conjugated secondary antibody. The assay result showed dose-dependent colorimetric signals for N-GFP (Figure 8A). In contrast, N-GFP Y109A, which was confirmed to be expressed at a comparable level to N-GFP and detectable with an anti-N protein antibody (Supplementary Figure S6), gave no signals. Importantly, the capturing of either N-GFP or N-GFP Y109A was not detectable when the T6-to-A variant of A58-20 DNA was used. These results demonstrate that the detection of N-GFP in this assay depends on the sequence-specific interaction between SARS-CoV-2 N protein and the DNA aptamer rather than non-specific nucleic acid-binding of the N protein. Based on a standard curve generated using a titration series of the purified N protein from *E. coli*, we estimated the N-GFP protein concentration in the 293T cell lysate to be 1.8 μg ml^−1^ (∼40 nM). A 320-fold diluted lysate (5.6 ng ml^−1^, ∼125 pM) gave a colorimetric signal at 3.5 times the background (Figure 8B), showing the sensitivity of this assay.

### Aptamer-mediated characterization of N-NTD RNA interaction

**Figure 9.**
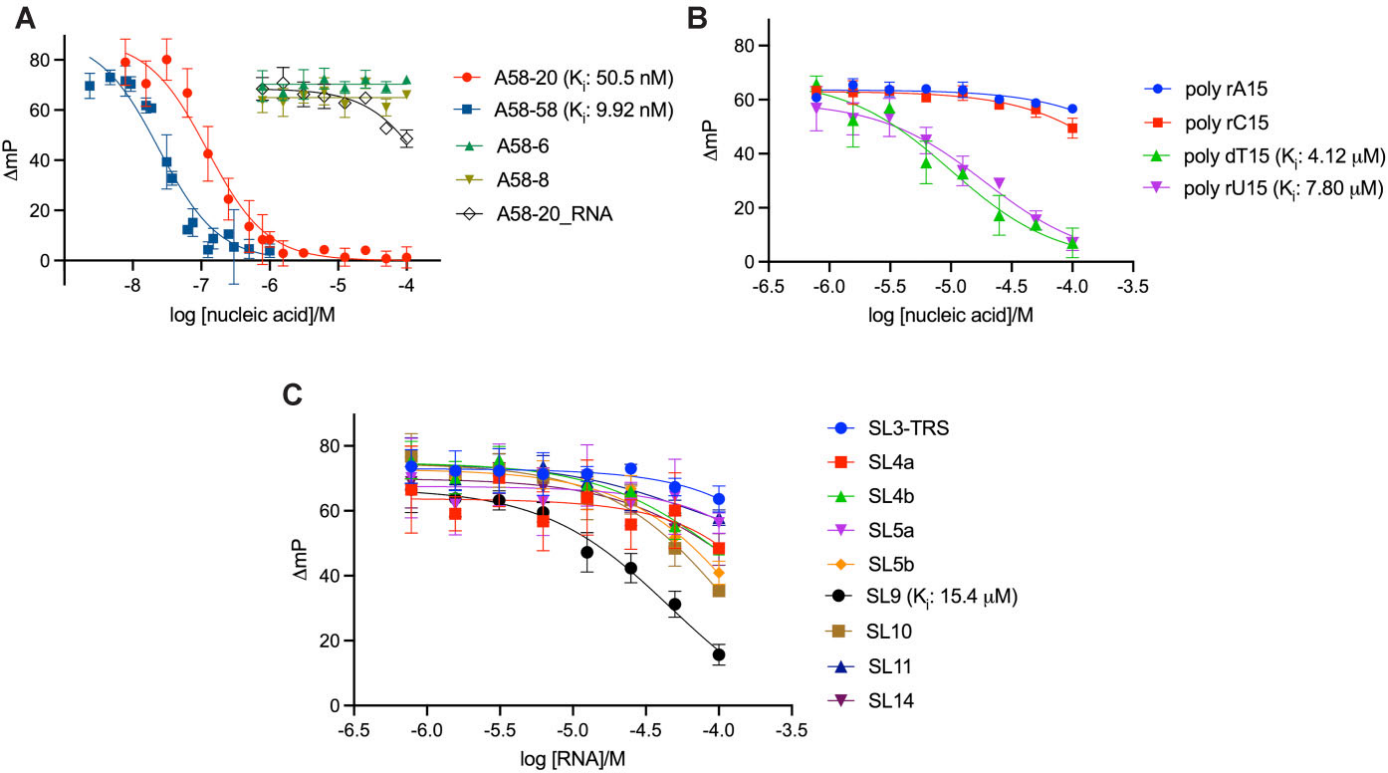
Competitive fluorescence anisotropy assay showing varying affinities of DNA or RNA oligonucleotides to N-NTD. (**A**) Dose-response curves for unlabeled A58-58, A58-20, A58-8 and A58-6 DNAs and A58-20 RNA displacing the fluorescein-labeled probe (A58-20-2′FG3-iT6FAM) from N-NTD. (**B**) Dose-response curves for poly-rA, poly-rC, and poly-rU RNA and poly-dT DNA displacing the labeled probe from N-NTD. (**C**) Dose-response curves for SARS-CoV-2 genome-derived RNA stem-loop motifs displacing the labeled probe from N-NTD. Data points are the mean ± standard deviation of three replicates. Individual graphs are shown in Supplementary Figure S7 and the estimated *K*_i_ values are summarized in Supplementary Table S2.

We further explored the utility of DNA aptamers in investigating the N-NTD interaction with various nucleic acids. As mentioned above, the internally modified aptamer, A58-20-2′FG3-iT6FAM, showed the highest affinity for N-NTD and greater fluorescence polarization signals when bound by N-NTD in our FA assay (Figure 5B). Thus, we used this fluorescently labeled oligonucleotide as a probe and examined N-NTD binding to various unlabeled oligonucleotides in a competition mode (Figure 9). First, we tested an unlabeled A58-20 DNA as a competitor and obtained an inhibition constant (*K*_i_) of 50.5 nM (95% CI: 38.5 – 66.2 nM) comparable to the K_D_ determined by FA in the direct binding mode, which validates this method for affinity estimation (Figure 9A, Supplementary Figure S7). The 58-nt A58 showed a slightly stronger inhibition (*K*_i_ = 9.92 nM, 95% CI: 7.68–12.9 nM). No inhibition was observed by A58-8 or A58-6, consistent with their poor affinity (Supplementary Figure S4). A58-20_RNA showed partial inhibition at higher concentrations tested, indicating weak but detectable affinity (*K*_i_ > 68 μM, Figure 9A, Supplementary Figure S7). Aside from variants of A58, we tested the binding of 15-nt DNA (poly-dT) or RNA (poly-rU, poly-rC, and poly-rA) homo-polymers and found that poly-dT and poly-rU have a much higher affinity for N-NTD (*K*_i_ = 4.12 and 7.80 μM, respectively) than poly-rC or poly-rA (*K*_i_ > 200 μM) (Figure 9B, Supplementary Figure S7). We then applied this assay to a panel of RNA oligonucleotides representing the RNA stem-loop motifs from the SARS-CoV-2 genome. Although these RNA oligonucleotides are expected to have weaker affinities based on their lack of co-elution with N-NTD in SEC (Supplementary Figure S2), they showed varying potency of inhibition (Figure 9C, Supplementary Figure S7). The best binder was SL9 with a *K*_i_ of 15.4 μM (95% CI: 12.2–19.6 μM), followed by SL10 (*K*_i_ = 38.7 μM, 95% CI: 30.8–49.5 μM). These results demonstrate the utility of an A58-20-derived DNA aptamer probe in investigating nucleic acid interaction of N-NTD and reveal a previously unappreciated sequence preference of N-NTD. The *K*_i_ values estimated using the competitive FA assay are summarized in Supplementary Table S2.

## Discussion

Building on work by Zhang *et al.* of SARS-CoV-2 N protein-binding DNA aptamers using SELEX (36), we identified a minimal DNA stem-loop motif that selectively binds to N-NTD. Structural analysis showed that the motif is centered on a hexanucleotide loop (5′-TCGGAT-3′), which fits in the highly basic groove of N-NTD and engages key RNA-binding residues. Key contacts include the bidentate hydrogen bonds with the cytosine base at the 2nd position by Arg92, and π-stacking interaction with the flipped-out thymine base at the 6th position as well as hydrogen bonding with a backbone phosphate by Tyr109 (Figure 4). R92E and Y109A mutations both abolish N-NTD binding to RNA (13,37), suggesting that our structure recapitulates aspects of N-NTD interaction with RNA. The pocket surrounded by Tyr109, Ser51, Arg88 and Tyr111 is of particular interest as earlier structural studies have shown a mononucleotide (AMP) and small molecule (PJ34) binding to an analogous position in N-NTD from another betacoronavirus, human CoV-OC43 (40), which suggests that this could be a conserved nucleotide-binding site. Studies have shown preferential binding of N-NTD to stem-loop motifs from the viral genome (41,42) and that stem-loop-containing RNAs promote RNP formation (17,43), suggesting the importance of N protein interaction with structured RNA in genome packaging. It is possible that the observed DNA stem-loop recognition mimics a mode of physiologically relevant RNA interaction, perhaps in the recognition of packaging signal sequences on the viral genome, as proposed for the guanine-specific binding by N-CTD (44). While the DNA stem-loop binding is mediated by N-NTD, N-CTD and/or IDRs may contribute to the binding to less structured nucleic acids, which could explain why the shorter A58-10 aptamer bound more tightly to full-length N protein than to N-NTD in the FA assay (Figure 2A versus C). The RNA-binding of full-length N protein in genome packaging may involve cooperative interaction of different regions of the protein to various uridine and guanosine-containing motifs.

We have demonstrated that the A58-20 DNA aptamer can be used in selective enrichment and detection of SARS-CoV-2 N protein from crude samples. DNA aptamers could complement protein-based antibodies in antigen detection assays, particularly because of superior thermal and chemical stability and ease of large-scale production. Even though the utility of DNA aptamers in SARS-CoV-2 antigen detection has been shown in earlier studies (36,45–48), the mechanism of their target recognition remained unknown. Our studies provide a structural explanation for a DNA aptamer targeting the N protein and reveal the binding epitope, which is instrumental in adapting the aptamer for diagnostic applications. As the SARS-CoV-2 N protein is the most common target of rapid antigen tests as the most abundant viral protein, mutations in new variants could compromise detection by altering the epitopes for antibodies (49). The specific engagement of functionally important residues by A58-20 may make it less susceptible to virus evolution. Although unlikely to be directly useful for clinical applications requiring a sub-ng ml^−1^ limit of detection (LoD) for the N protein antigen, the ELISA assay we developed (Figure 8) would be sensitive enough to detect N protein in samples from higher-titer patients, which can reach several μg ml^−1^ (4,7). The aptamer-mediated pull-down (Figure 7) could also be particularly useful in concentrating the antigen from dilute and large-volume samples in diagnostic applications.

The engagement of the RNA-binding groove of SARS-CoV-2 N protein by A58-20 makes this DNA aptamer a useful probe for assay development, especially given that the amino acid sequence of N-NTD is nearly 100% conserved across all variants of SARS-CoV-2. As a proof of principle, we showed that a chemically modified A58-20 variant, A58-20-2′FG3-iT6FAM, can be used to evaluate the affinities of various unlabeled nucleic acids to N-NTD. This competition-based assay enabled us to uncover a previously unappreciated sequence preference of N-NTD. The observed higher affinity for poly-dT and poly-rU over poly-rC and poly-rA is possibly a result of interaction with the thymine/uracil-binding pocket identified in our crystallographic studies (Figure 4) and suggests that uridine-rich RNAs would be preferentially bound by N-NTD. SL9, which showed the highest affinity among the viral stem-loop motifs tested (Figure 9) is indeed uridine-rich, with 7 out of 15 nucleotides being uridines. However, the second-best binder, SL10, contains only 4 uridines out of 15 nucleotides, which is the same as the poorest binder, SL3-TRS. Thus, sequence context is likely to play a role in dictating the affinity beyond the simple uridine content. The observed weakest binding of SARS-CoV-2 N-NTD to SL3-TRS is notable, as it contrasts with the preferential binding of MHV N-NTD to SL3-TRS reported earlier (26). Our studies will facilitate further investigations into RNA-binding mechanisms of the SARS-CoV-2 N protein and may aid in the future development of novel diagnostic or therapeutic strategies.

## Supplementary Material

gkae874_Supplemental_File

## Data Availability

Atomic coordinates and structure factors have been deposited in the Protein Data Bank (PDB) under the accession code 8TFD. All other data are available from the authors upon request.

## Notes

Present address: Seyed Arad Moghadasi, NYU School of Medicine, New York, NY 10016, USA.
