# Supplementary material for: A compact stem-loop DNA aptamer targets a uracil-binding pocket in the SARS-CoV-2 nucleocapsid RNA-binding domain
Source: Nucleic Acids Res. 2024 Oct 9;52(21):13138–51. doi: 10.1093/nar/gkae874 (PMC11602162; doi:10.1093/nar/gkae874)
Supplement: gkae874_Supplemental_File [file gkae874_supplemental_file.pdf]

Supplementary information

A compact stem-loop DNA aptamer targets a uracil-binding pocket in the SARS-CoV-2 nucleocapsid RNA-binding domain

Esler, Belica *et al.*

This PDF file includes:

Figure S1: SEC binding analyses of various DNA and RNA aptamers to N-NTD or N-CTD p.2 - 8

Figure S2: SEC binding analyses of viral stem-loop RNA motifs to N-NTD p.9 - 11

Figure S3: SEC binding analyses of A58 aptamer derivatives to N-NTD (co-elution observed) p.12

Figure S4: SEC binding analyses of A58 aptamer derivatives to N-NTD (co-elution not observed) p.13

Figure S5: Electron density map for the N-NTD / A58-20 complex p.14

Figure S6: Western blot of N-GFP and N-GFP Y109A proteins p.15

Figure S7: Competitive binding of DNA and RNA motifs to N-NTD (individual graphs for data in Fig. 9) p.16

Table S1: Summary of K_D values measured using fluorescence anisotropy p.17

Table S2: Summary of K_i values estimated using a competition-based fluorescence anisotropy assay p.18

DNA aptamer A15 from Zhang et al., *Chem. Commun.* 2020, 56, 10235-10238
GCTGGATGTTCATGCTGGCAAAATTCCTTAGGGGCACCGTTACTTTGACACATCCAGC

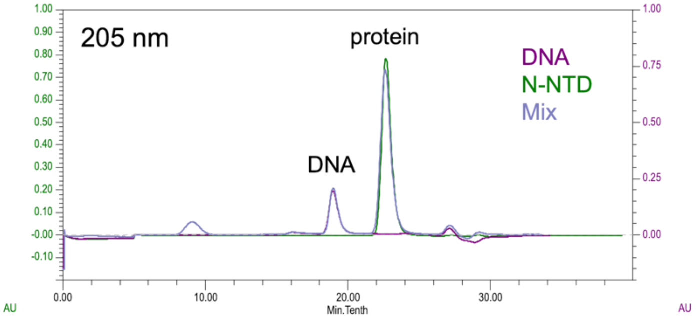

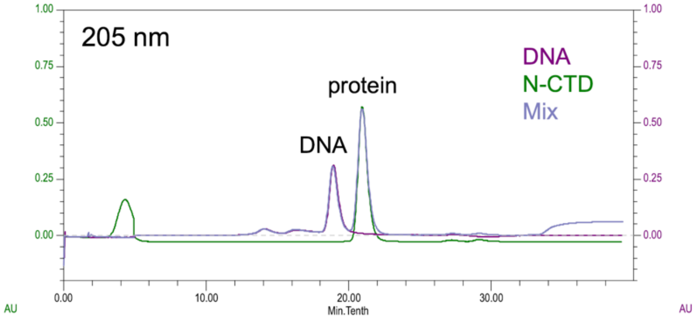

DNA aptamer A15

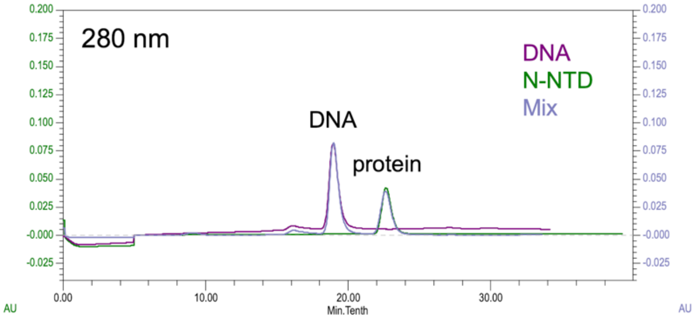

DNA aptamer A15

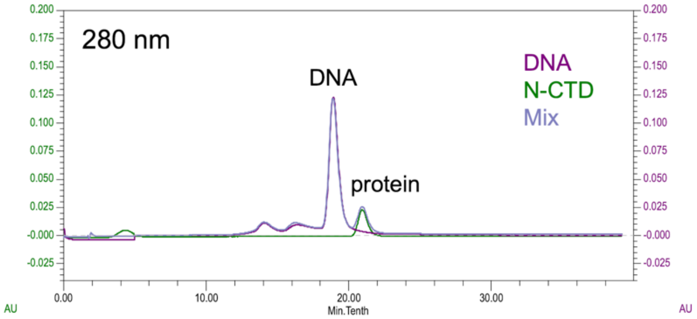

DNA aptamer A48 from Zhang et al., *Chem. Commun.* 2020, 56, 10235-10238
GCTGGATGTCGCTTACGACAATATTCCTTAGGGGCACCGCTACATTGACACATCCAGC

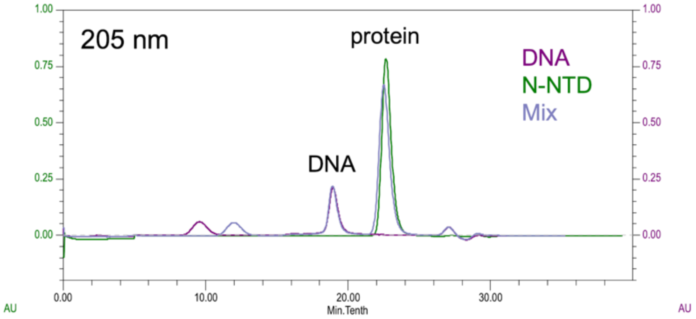

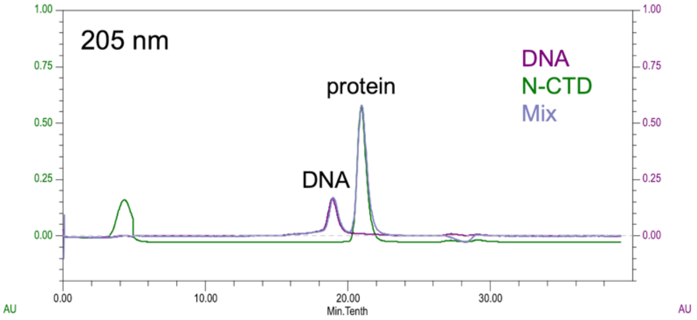

DNA aptamer A48

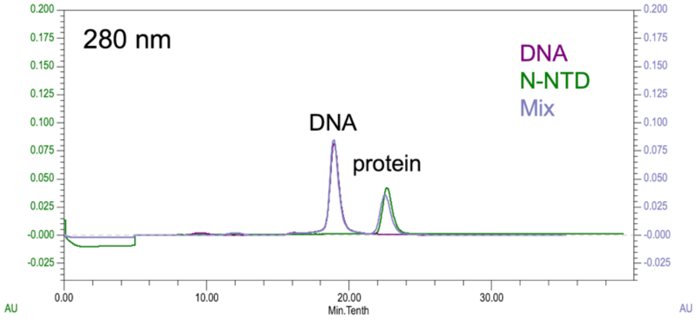

DNA aptamer A48

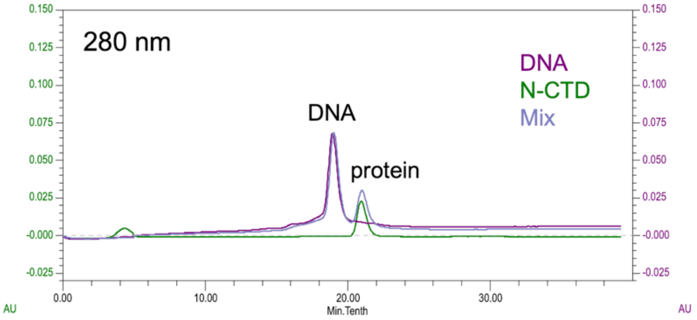

DNA aptamer A58 from Zhang et al., *Chem. Commun.* 2020, 56, 10235-10238
GCTGGATGTCACCGGATTGTCGGACATCGGATTGTCTGAGTCATATGACACATCCAGC

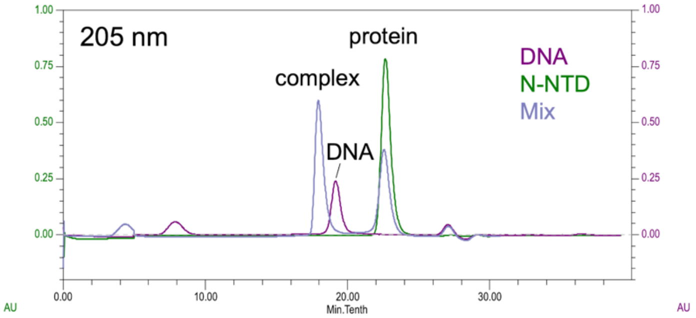

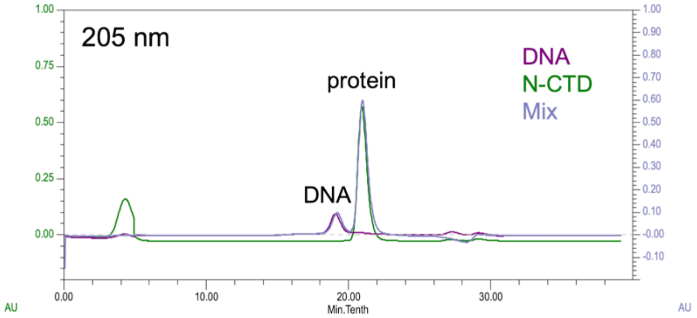

DNA aptamer A58

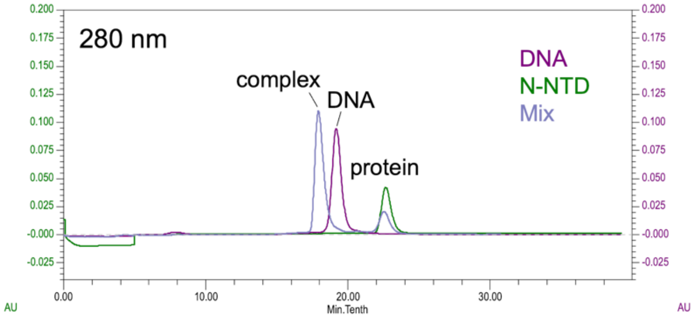

DNA aptamer A58

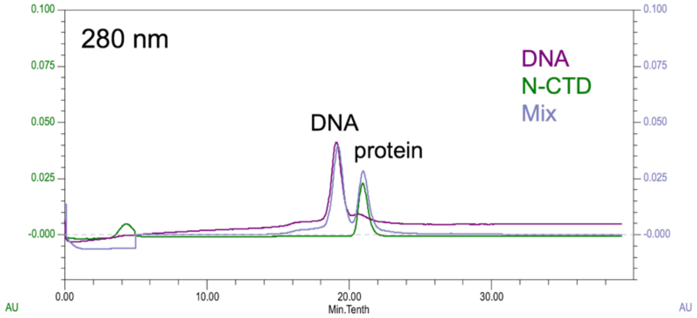

DNA aptamer A61 from Zhang et al., *Chem. Commun.* 2020, 56, 10235-10238
GCTGGATGTTGACCTTTACAGATCGGATTCTGTGGGGCGTTAAACTGACACATCCAGC

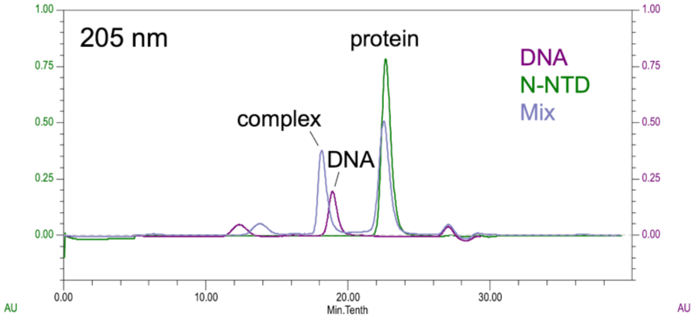

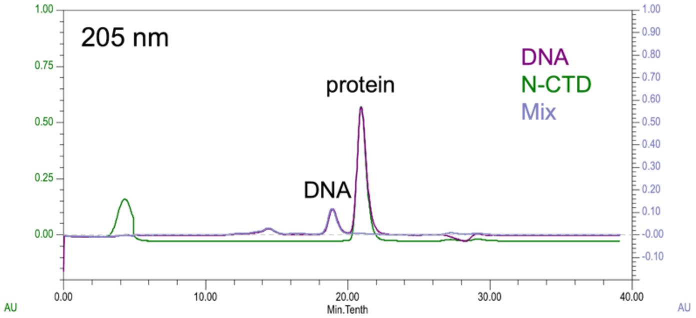

DNA aptamer A61

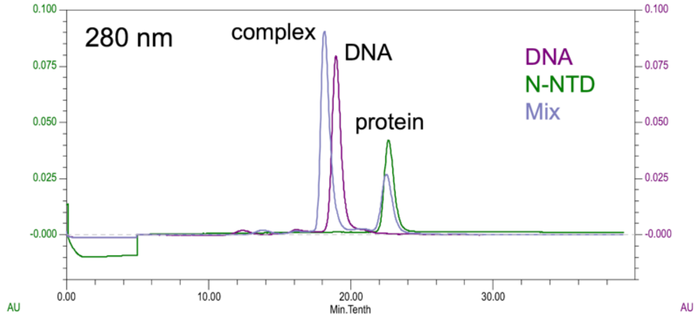

DNA aptamer A61

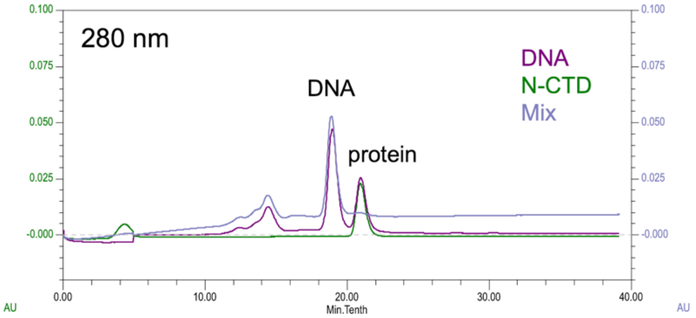

DNA aptamer 3 from Chen et al., *Virol. Sin.* 2020, 35, 351-354
GCAATGGTACGGTACTTCCGGATGCGGAAACTGGCTAATTGGTGAGGCTGGGGCGGT

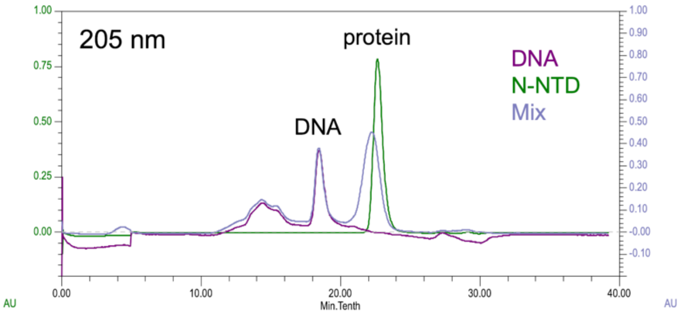

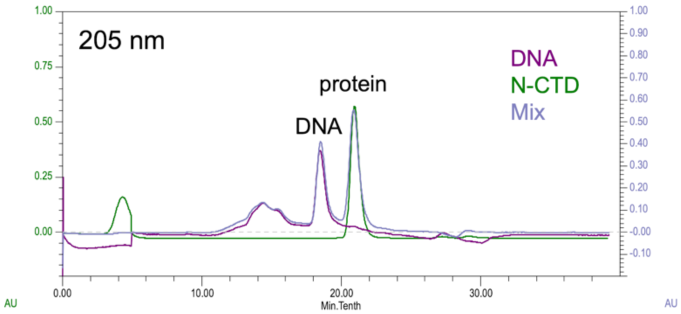

DNA aptamer 3

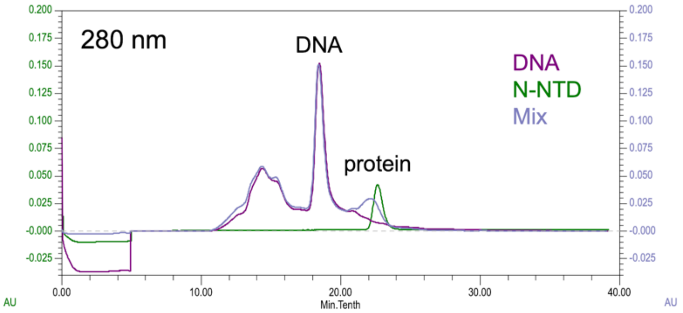

DNA aptamer 3

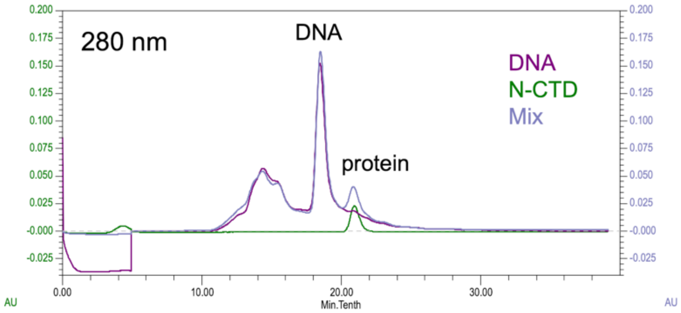

DNA aptamer 11 from Cho et al., *J Biosci Bioeng.* 2011, 112, 535-540
GCAATGGTACGGTACTTCCCCGTAGATCGAGGGAGCGCATTAAGGTATACGCCCTTCCCATCTTCAAAAGTGCACGCTACTTTGCTAA

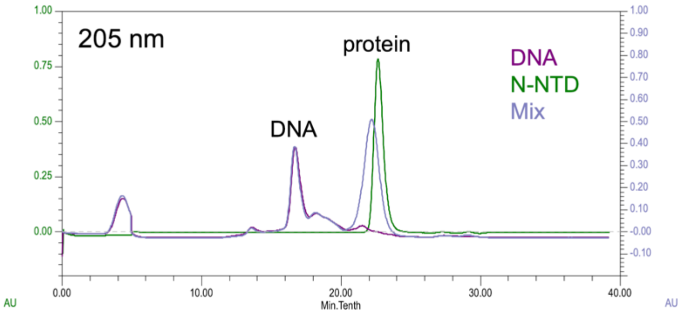

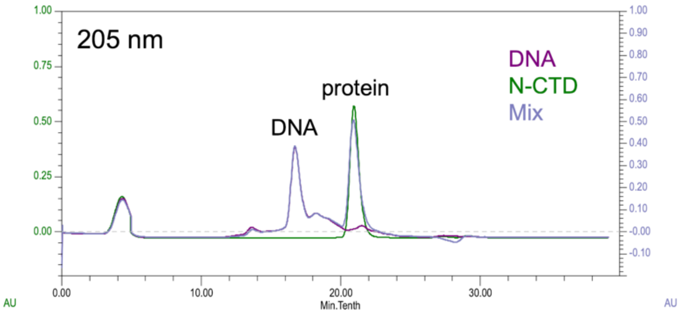

DNA aptamer 11

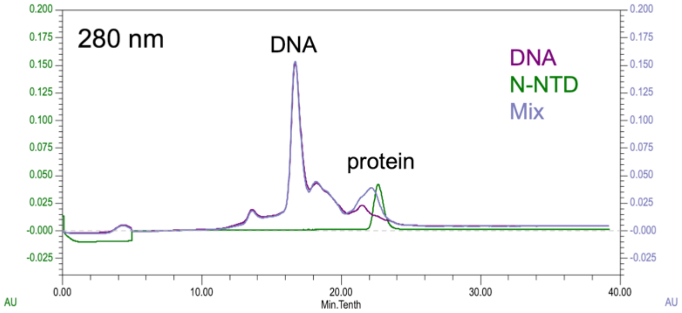

DNA aptamer 11

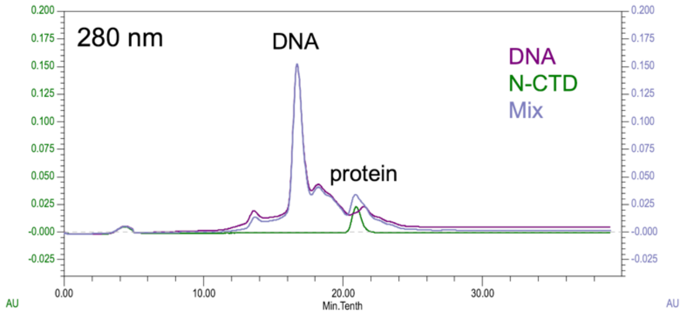

DNA aptamer from Kang et al., *Anal. Chem.* 2021, 93, 992-1000
ATCCAGAGTGACGCAGCAAACCCAAGCAAACTACCTCTATACCCTTCGACCTTCATCATGGACACGGTGGCTTAGT

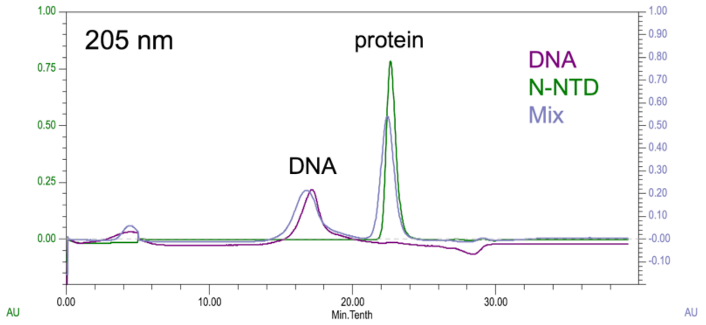

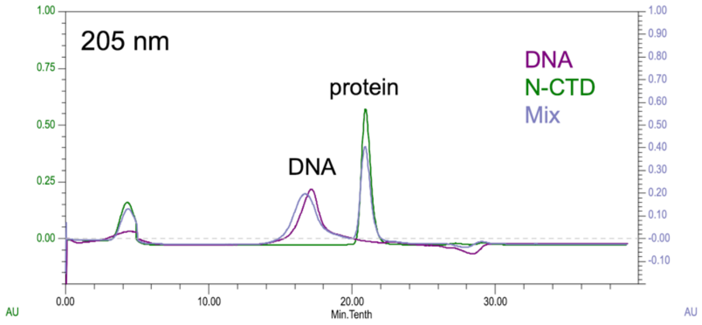

DNA aptamer from Kang et al.

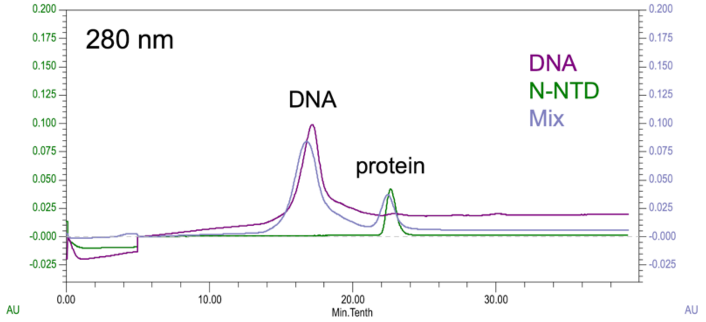

DNA aptamer from Kang et al.

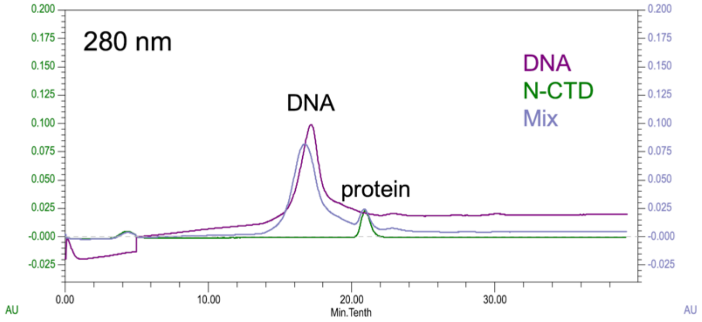

DNA aptamer 1 from Cho et al., *J Biosci Bioeng.* 2011, 112, 535-540
GCAATGGTACGGTACTTCCGGATGCGGAAACTGGCTAATTGGTGAGGCTGGGGCGGTCGTGCAGCAAAAGTGCACGCTACTTTGCTAA

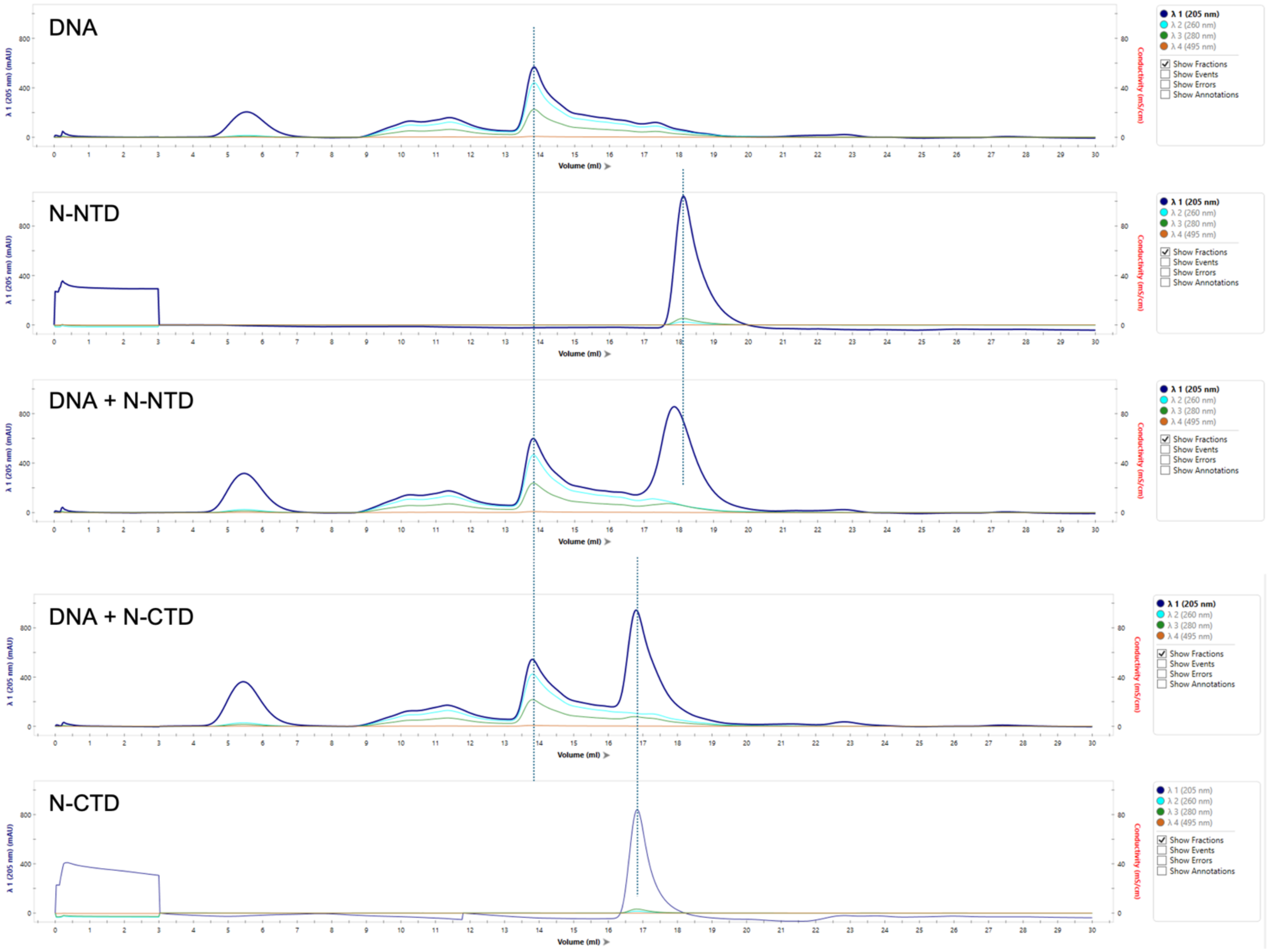

RNA aptamer 1 from Ahn et al., *Analyst.* 2009, 134, 1896–1901
rUrGrUrCrGrUrUrCrGrCrUrGrUrCrUrUrGrCrUrArCrGrUrUrArCrGrUrUrArCrArCrGrGrUrUrGrGrCrA

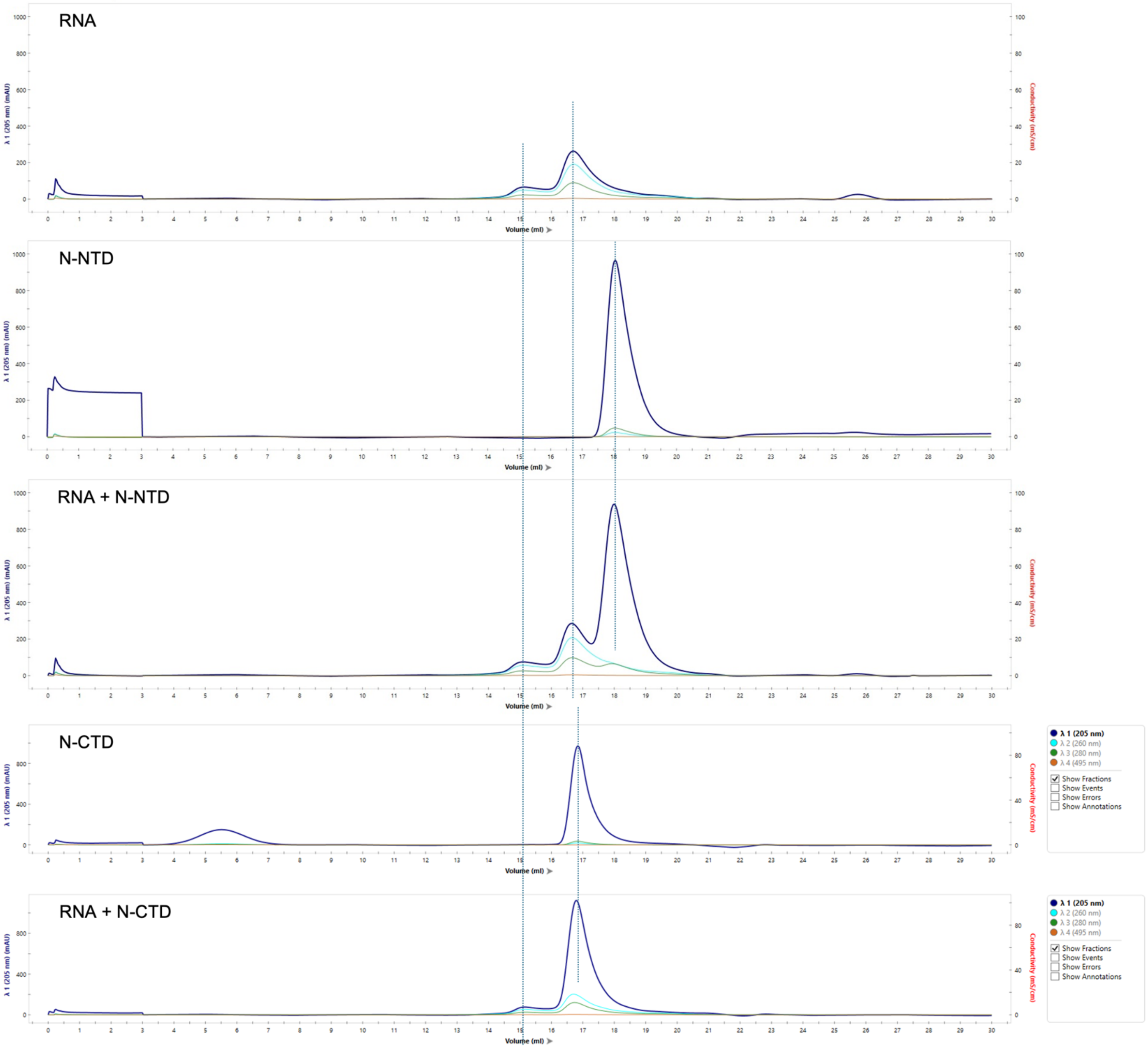

```
RNA aptamer 2 from Ahn et al., Analyst. 2009, 134, 1896–1901
rUrCrArUrUrArCrArCrArCrArUrCrUrCrArCrGrGrGrArGrArCrArUrArGrCrUrGrArCrGrArUrArUrCrCrA
```

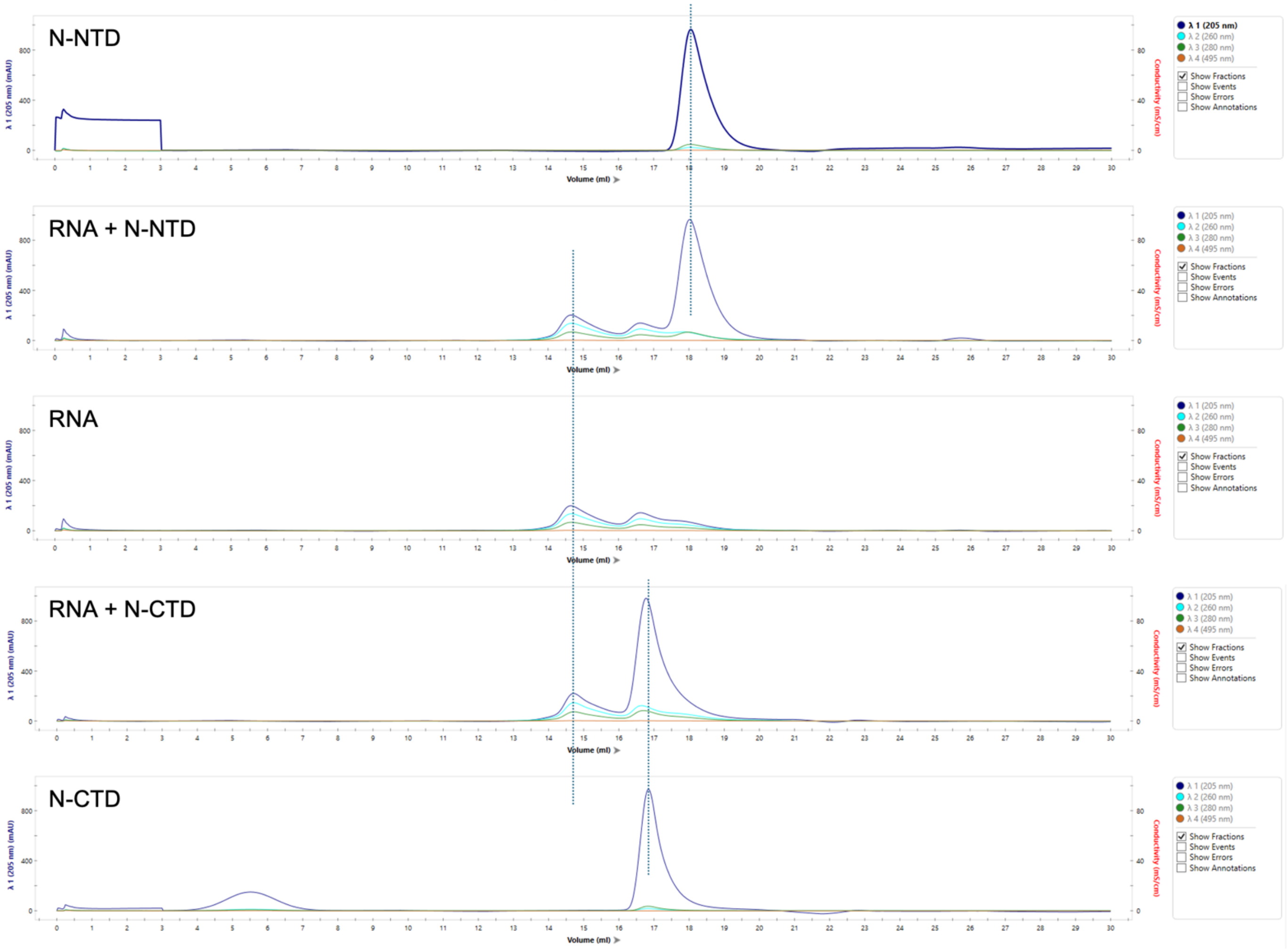

Figure S1. Size-exclusion chromatography (SEC) binding analyses of various DNA and RNA aptamers to N-NTD or N-CTD

Overlaid chromatograms for individual (protein or nucleic acid alone) injections and their co-injection into a Superdex 200 10/300 column, monitored at multiple wavelengths. For data in pages 2 ~ 5, the flow rate was 0.75 mL min^{-1}.

RNA SL3-TRS
rGrUrUrCrUrCrUrArArArCrGrArArC

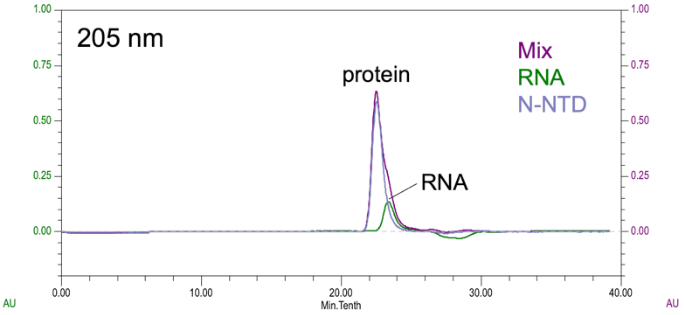

RNA SL3-TRS

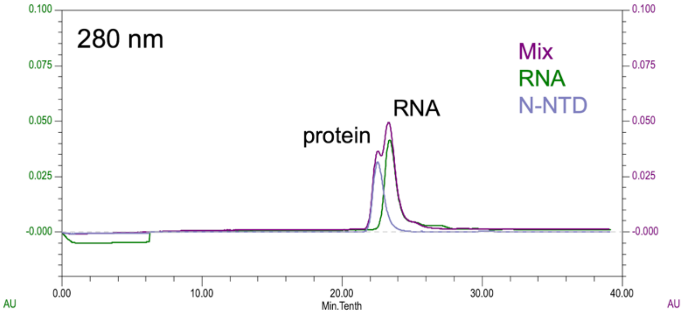

RNA SL4a
rGrGrCrUrGrCrArUrGrCrU

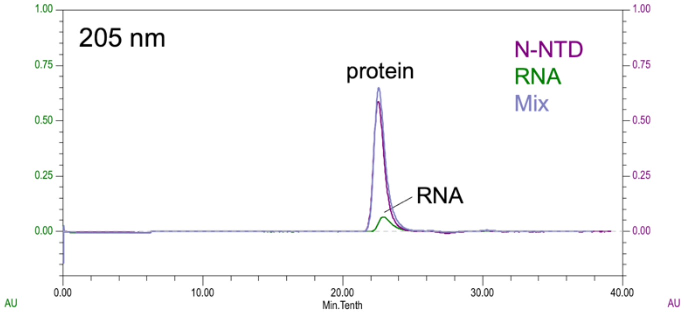

RNA SL4a

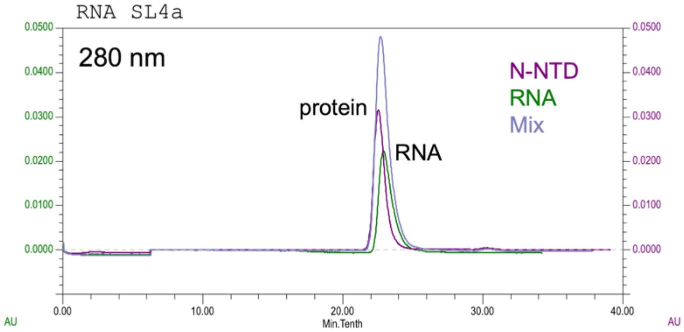

RNA SL4b
rUrArArUrArArCrUrArArUrUrA

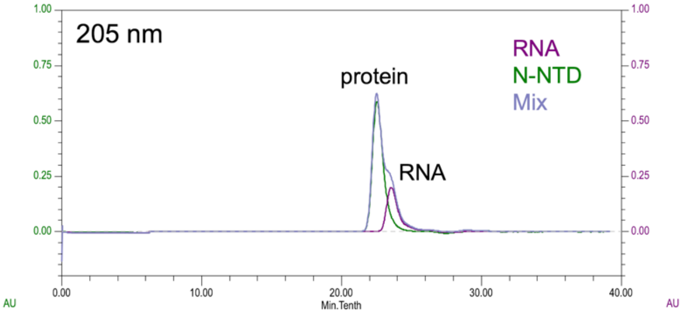

RNA SL4b

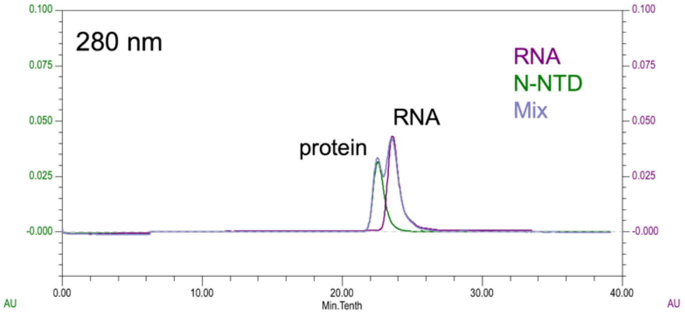

RNA SL5a
rArCrGrGrUrUrUrCrGrUrCrCrGrU

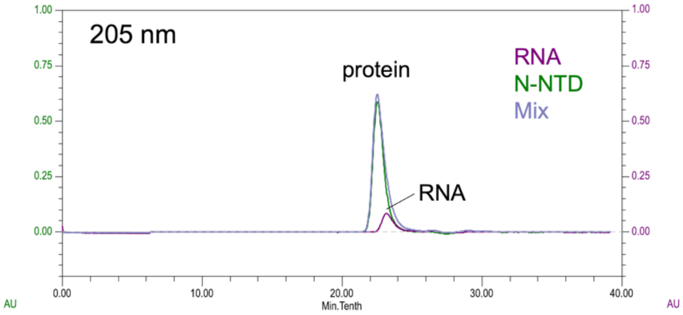

RNA SL5a

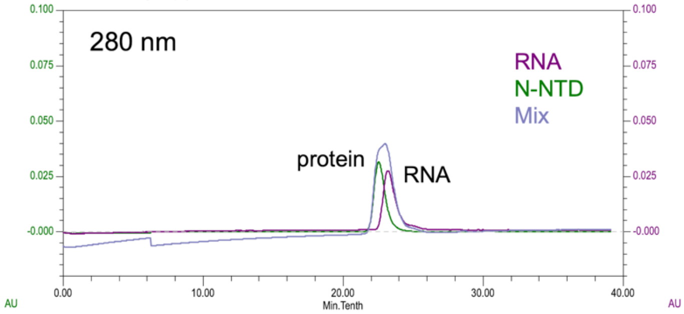

RNA SL5b
rCrUrArGrGrUrUrUrCrGrUrCrCrGrG

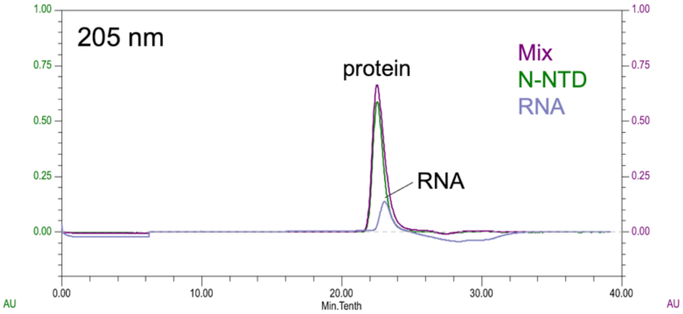

RNA SL5b

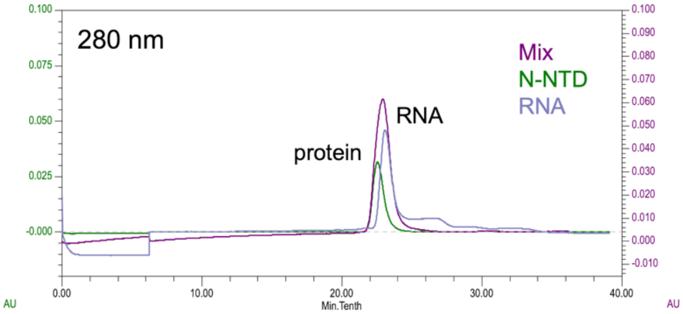

RNA SL9
rUrCrArUrGrUrUrArUrGrGrUrUrGrA

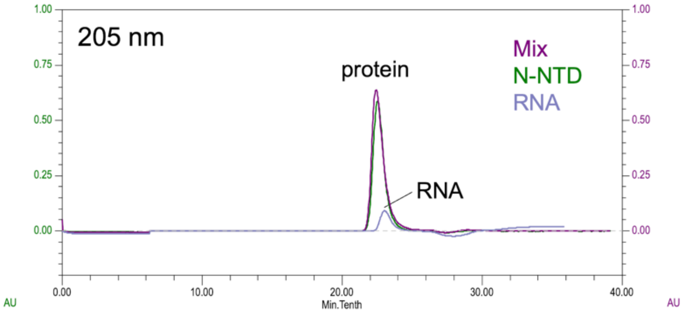

RNA SL9

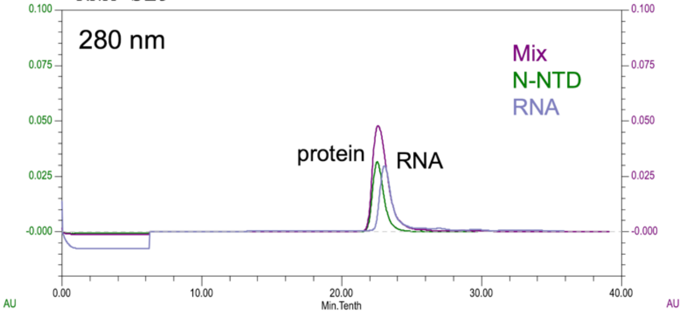

RNA SL10
rGrUrCrCrCrUrCrArUrGrUrGrGrGrC

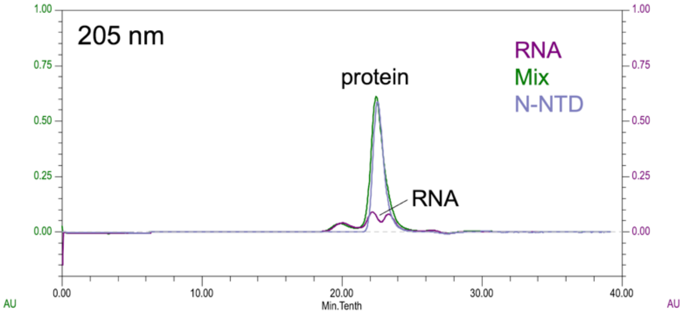

RNA SL10

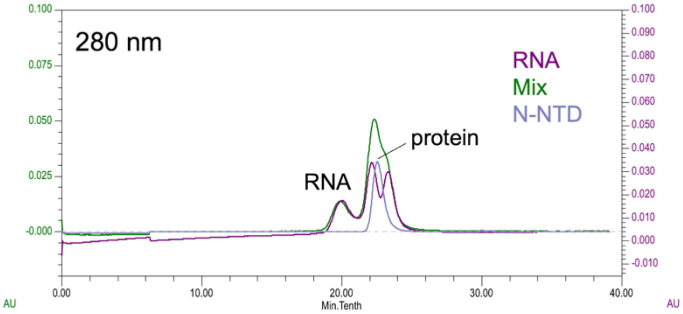

RNA SL11
rUrUrCrUrUrCrUrUrCrGrUrArArGrArA

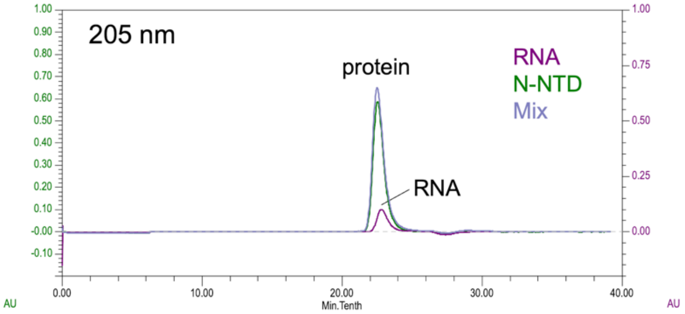

RNA SL11

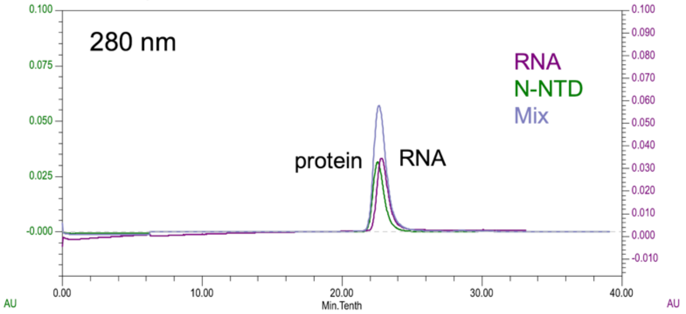

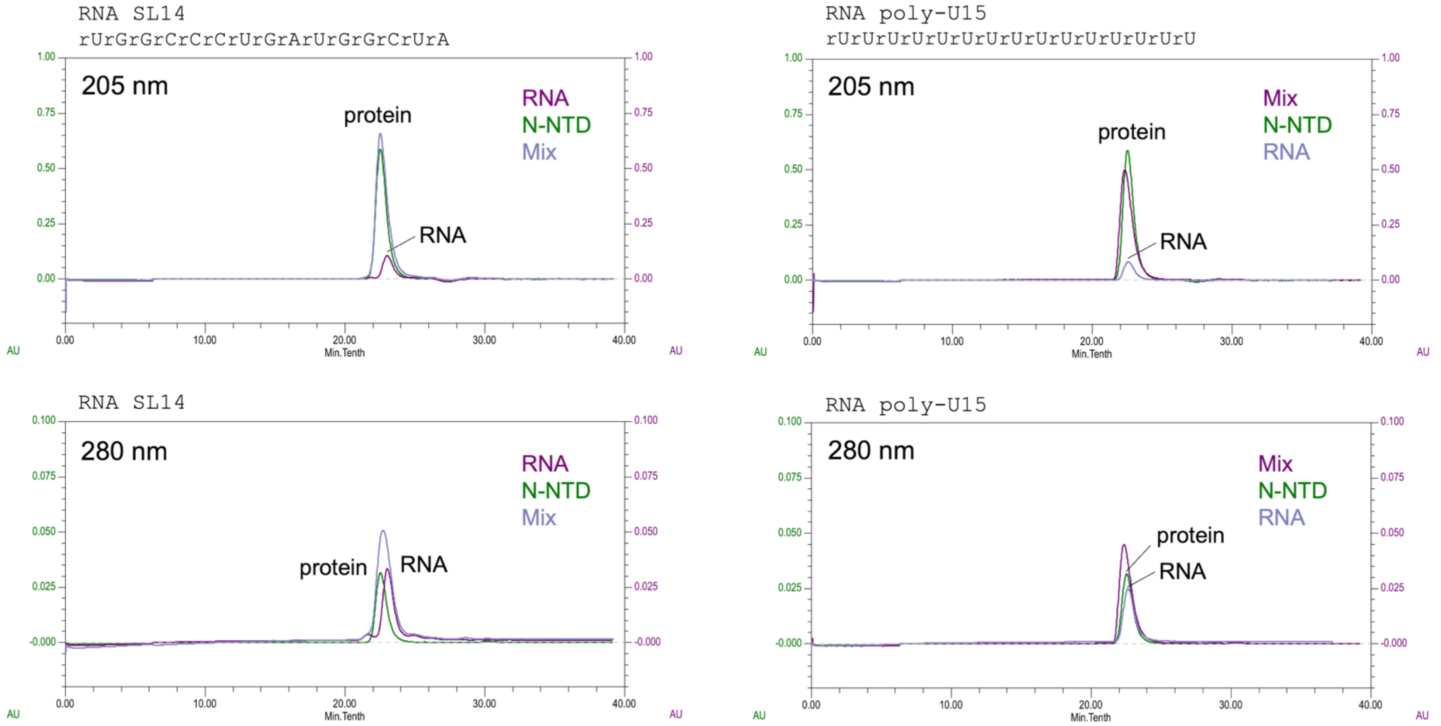

Figure S2. SEC binding analyses of viral stem-loop RNA motifs to N-NTD

Overlaid chromatograms for individual (protein or RNA alone) injections and their co-injection into a Superdex 200 10/300 column, monitored at two wavelengths. The flow rate was 0.75 mL min^{-1}. Although these RNA oligonucleotides did not form stable enough complexes with N-NTD detectable by co-elution in SEC, some of them showed modest affinity to N-NTD in the competitive fluorescence anisotropy assay (Fig. 9, Supplementary Figure S7, and Supplementary Table S2).

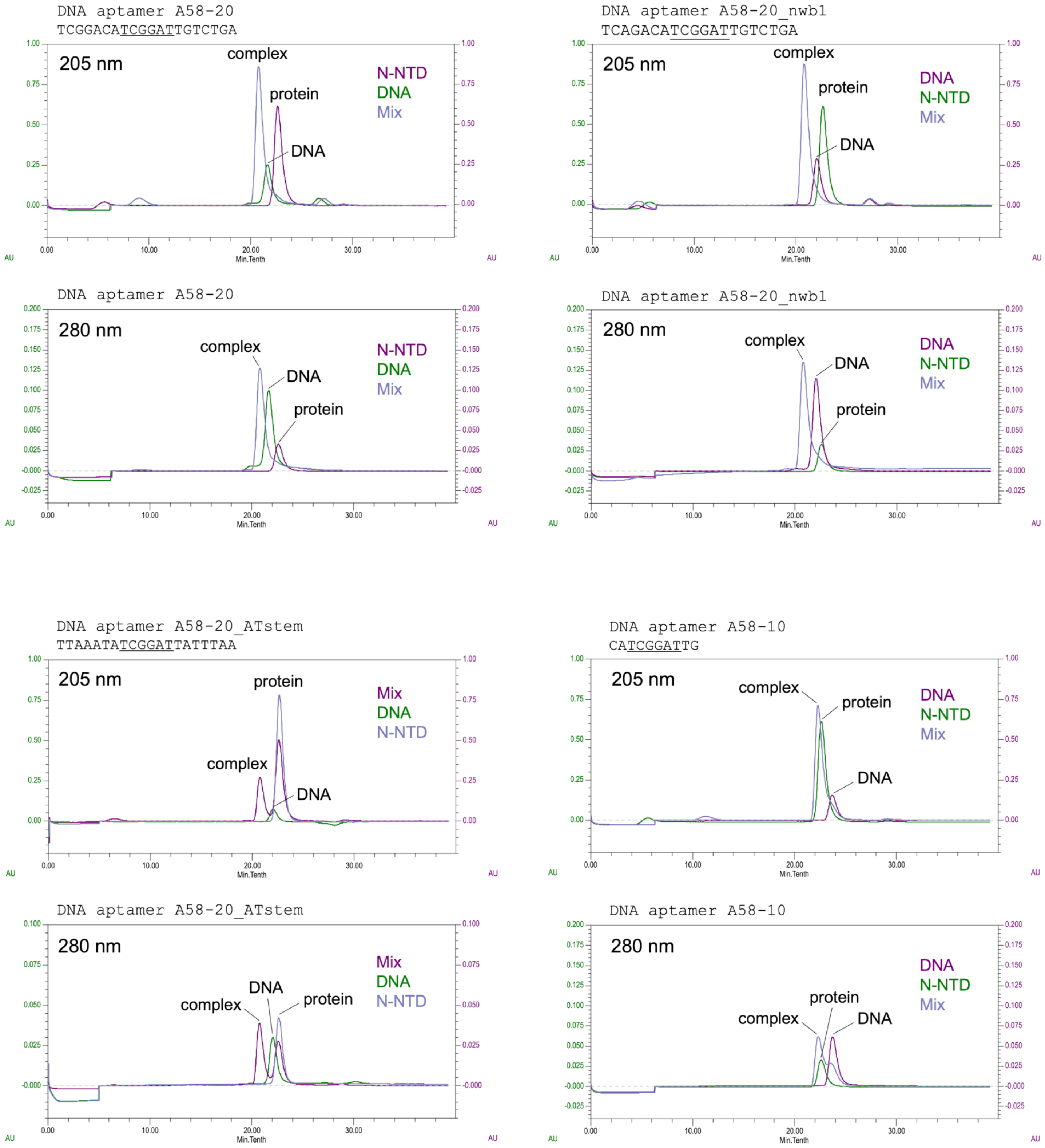

Figure S3. SEC binding analyses of A58 aptamer derivatives to N-NTD (co-elution observed)

Overlaid chromatograms for individual (protein or DNA alone) injections and their co-injection into a Superdex 200 10/300 column, monitored at two wavelengths. The flow rate was 0.75 mL min^{-1}. Note the co-elution of N-NTD-DNA complexes earlier than the protein or DNA alone.

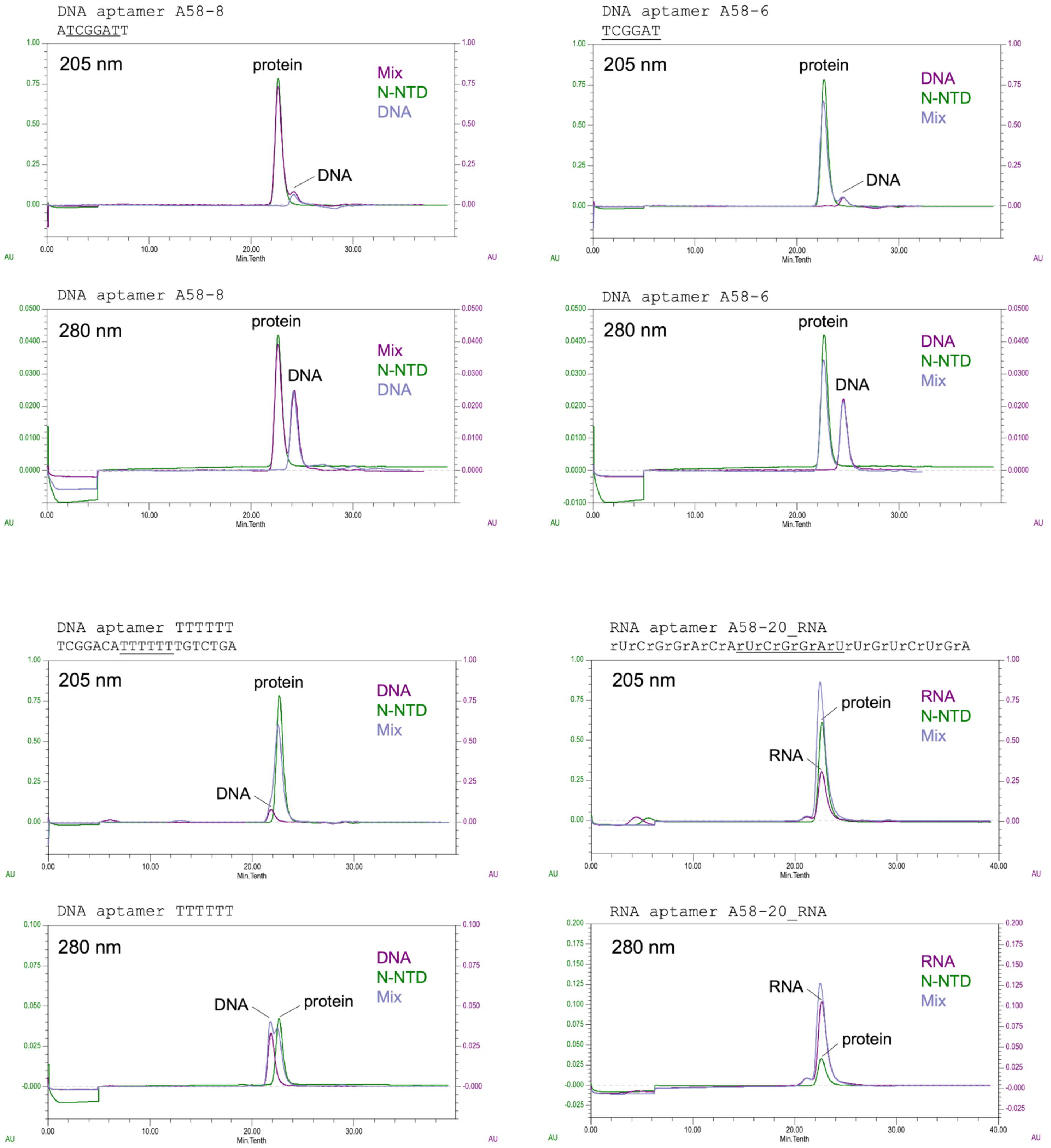

Figure S4. SEC binding analyses of A58 aptamer derivatives to N-NTD (co-elution not observed)

Overlaid chromatograms for individual (protein or nucleic acid alone) injections and their co-injection into a Superdex 200 10/300 column, monitored at two wavelengths. The flow rate was 0.75 mL min^{-1}.

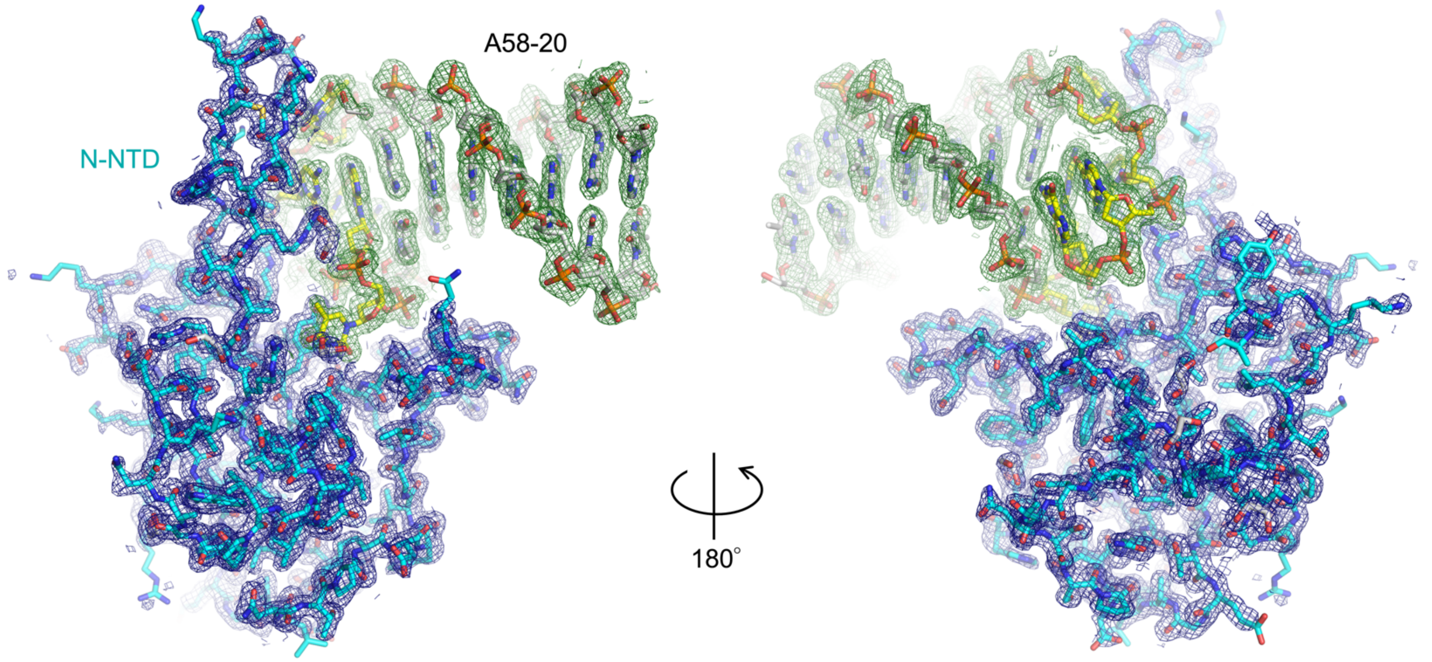

Figure S5. Electron density map for the N-NTD / A58-20 complex at 1.55-Å resolution

The 2mFo-DFc electron density map contoured at 1.0 σ is shown as a blue (within 2.2 Å from any atom in the protein) or green (within 2.2 Å from any atom in the DNA) mesh. The N-NTD and A58-20 molecules are shown as sticks, colored as in Fig. 4.

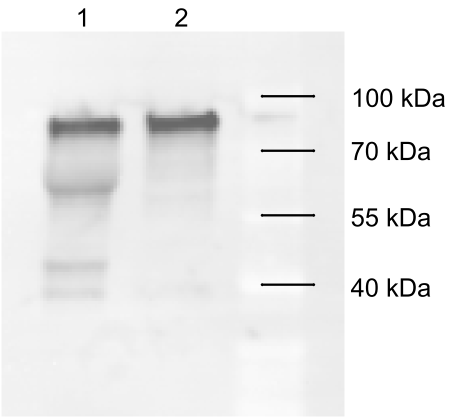

Figure S6. Western blot of N-GFP and N-GFP Y109A proteins

Whole cell lysate from 293T cells transfected with a plasmid DNA for expressing 1) N-GFP or 2) N (Y109A)-GFP was resolved by SDS-PAGE, transferred to a PVDF membrane and incubated with anti-N protein antibody 1:2500 (Sino Biological 40143-MM05) and LICOR IRDye 800CW anti-Mouse IgG 1:20,000 (Li-Cor 925-32210). The result shows comparable expression levels of the two proteins.

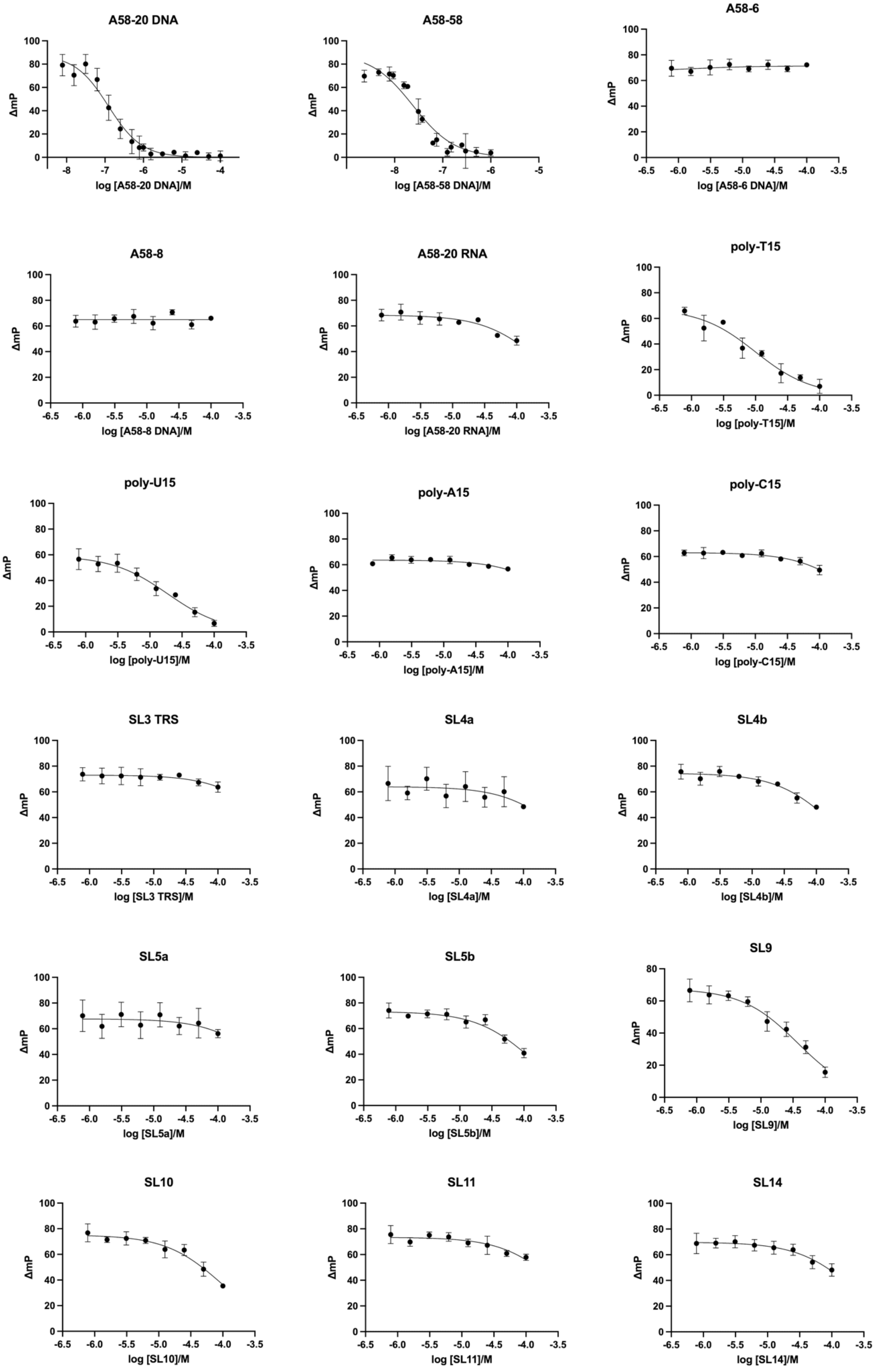

Figure S7: Competitive binding of DNA and RNA motifs to N-NTD (individual graphs for data in Fig. 9)

Supplementary Table S1

Summary of K_D values for N-NTD binding measured using fluorescence anisotropy

Name	Sequence	K_D	95% confidence interval
A58-20 (A58-20-3'FAM)	TCGGACATCGGATTGTCTGA/36-FAM/	101 nM	91.4 to 110
2'FG3	TCGGACATC/i2FG/GATTGTCTGA/36-FAM/	74.0 nM	60.9 to 89.4
A58_20_Nwb1	TCAGACATCGGATTGTCTGA/36-FAM/	84.9 nM	65.5 to 109
A58_20_Nwb2	TCGGACATCGGATTGTCCGA/36-FAM/	87.9 nM	72.0 to 107
A58-10	CATCGGATTG/36-FAM/	2.27 μM	1.91 to 2.69
TTTTTT	TCGGACATTTTTTTGTCTGA/36-FAM/	>6.1 μM	
T6-to-A	TCGGACATCGGAATGTCTGA/36-FAM/	Not determined	
C2-to-G	TCGGACATGGGATTGTCTGA/36-FAM/	Not determined	
NNNNNN	TCGGACANNNNNNTGTCTGA/36-FAM/	Not determined	
A58-20_RNA	rUrCrGrGrArCrArUrCrGrGrArUrUrGrUrC rUrGrA/36-FAM/	Not determined	
A58-20-iT6FAM	TCGGACATCGGA/i6-FAMK/TGTCTGA	74.0 nM	64.9 to 84.1
A58-20-2'FG3-iT6FAM	TCGGACATC/i2FG/GA/i6-FAMK/TGTCTGA	13.9 nM	10.0 to 18.8

The hexanucleotide loop region is underlined. 'N' denotes a random base. 'r' denotes a ribonucleotide.

36-FAM: 3'-fluorescein

i2FG: 2'-deoxy-2'-α-fluoroguanosine

i6-FAMK: 2'-deoxyuridine with fluorescein attached to the pyrimidine C5 position

Supplementary Table S2

Summary of K_i values estimated using a competition-based fluorescence anisotropy assay (Fig. 9 and Fig. S7)

Name	Sequence	K_i	95 % confidence interval
A58-20	TCGGACATCGGATTGTCTGA	50.5 nM	38.5 to 66.2
A58-58	GCTGGATGTCACCGGATTGTCGGACATCGGATTGTCTGAGTCATATGACACATCCAGC	9.92 nM	7.68 to 12.9
A58-6	TCGGAT	No inhibition	
A58-8	ATCGGATT	No inhibition	
A58-20_RNA	rUrCrGrGrArCrArUrCrGrGrArUrUrGrUrCrUrGrA	90.8 µM	67.7 to 129
poly-T15	TTTTTTTTTTTTTTT	4.12 µM	2.82 to 6.05
poly-U15	rUrUrUrUrUrUrUrUrUrUrUrUrUrUrU	7.80 µM	5.79 to 10.6
poly-A15	rArArArArArArArArArArArArArArA	>200 µM	
poly-C15	rCrCrCrCrCrCrCrCrCrCrCrCrCrCrC	153 µM	119 to 209
SL3-TRS	rGrUrUrCrUrCrUrArArArCrGrArArC	283 µM	169 to 713
SL4a	rGrGrCrUrGrCrArUrGrCrU	144 µM	66.3 to 981
SL4b	rUrArArUrArArCrUrArArUrUrA	69.7 µM	55.6 to 90.0
SL5a	rArCrGrGrUrUrUrCrGrUrCrCrGrU	226 µM	>93
SL5b	rCrUrArGrGrUrUrUrCrGrUrCrCrGrG	53.7 µM	43.0 to 68.6
SL9	rUrCrArUrGrUrUrArUrGrGrUrUrGrA	15.4 µM	12.2 to 19.6
SL10	rGrUrCrCrCrUrCrArUrGrUrGrGrGrC	38.7 µM	30.8 to 49.5
SL11	rUrUrCrUrUrCrUrUrCrGrUrArArGrArA	131 µM	92.2 to 207
SL14	rUrGrGrCrCrCrUrGrArUrGrGrCrUrA	86.4 µM	63.1 to 127

'r' denotes a ribonucleotide.
